# SHIFTPLAN: a randomized controlled trial investigating the effects of a multimodal shift-work intervention on drivers’ fatigue, sleep, health, and performance parameters.

**DOI:** 10.1186/s13063-022-06573-6

**Published:** 2022-08-17

**Authors:** Inge Declercq, Filip Van Den Eede, Ella Roelant, Johan Verbraecken

**Affiliations:** 1 Multidisciplinary Sleep Disorders Center, Antwerp University Hospital (UZA), Drie Eikenstraat 655 - 2650, Edegem, Belgium; 2 Department of Psychiatry, Antwerp University Hospital (UZA), Drie Eikenstraat 655 - 2650, Edegem, Belgium; 3 Collaborative Antwerp Psychiatric Research Institute (CAPRI), Faculty of Medicine and Health Sciences, University of Antwerp (UA), Universiteitsplein 1, 2610 Antwerp, Belgium; 4 Clinical Trial Center (CTC), CRC Antwerp, Antwerp University Hospital (UZA), University of Antwerp, Edegem, Belgium; 5 Medical Coordinator Multidisciplinary Sleep Disorders Center (UZA), LEMP FGGW UA, Antwerp University Hospital (UZA), Drie Eikenstraat 655 - 2650, Edegem, Belgium

**Keywords:** Shift work, Shift work disorder, Fatigue, Insomnia, Health, Sleep, Chronotype, Intervention, Education, Scheduling

## Abstract

**Background:**

Shift work is commonly associated with health problems resulting from circadian misalignment and sleep restriction. About one in three shift workers is affected by insomnia and up to 90% report regular fatigue and/or sleepiness at the workplace. Epidemiological data shows that shift workers are at increased risk of cardiovascular disease, diabetes, obesity, breast cancer, mental-health problems, and shift-work disorder, which conditions typically lead to reduced work performance, processing errors, accidents at work, absenteeism, and reduced quality of life. Given these widespread and debilitating consequences, there is an urgent need for treatments that help improve the sleep, health, and functional performance of the shift-working population. The most common non-pharmacological recommendations are improved scheduling, bright-light exposure, napping, psychoeducation promoting sleep hygiene, and cognitive-behavioral techniques. The objectives of the present study are to investigate the effects of a multimodal shift-work intervention on perceived fatigue, sleepiness, physical and mental health, sleep parameters, and absenteeism.

**Methods:**

A randomized controlled interventional study comparing the two groups each comprising at least 80 drivers of a public transport company, using self-report questionnaires and health checks completed at intake and after 3 and 6 months following the start of the intervention or waiting-list period. The intervention consists of (a) healthy scheduling taking into account shift-rotation direction and speed, chronotype, resting time, and napping; (b) an education program specifically developed for shift workers; and (c) a dedicated information campaign for shift planners. The primary outcome is symptomatic burden in terms of sleepiness, and the key secondary outcome is symptomatic burden in terms of fatigue. Supplementary secondary outcomes are sleep parameters, absenteeism, general and clinical health, changes in mood, and anxiety.

**Discussion:**

Expected outcomes are significant improvements on all primary and secondary outcome parameters in the intervention group. To our knowledge, ours is the first randomized controlled study to systematically investigate the effects of a multimodal program on multiple health, sleep, and performance parameters in shift workers. Our research also aims at providing evidence-based practice guidelines for healthy scheduling in general and thus contribute to diminishing the serious health and economic burdens associated with shift work overall.

**Trial registration:**

EDGE registration number: 000339. ClinicalTrials.gov NCT05452096

**Supplementary Information:**

The online version contains supplementary material available at 10.1186/s13063-022-06573-6.

## Administrative information

Note: The numbers in curly brackets in this protocol refer to SPIRIT checklist item numbers. The order of the items has been modified to group similar items (see http://www.equator-network.org/reporting-guidelines/spirit-2013-statement-defining-standard-protocol-items-for-clinical-trials/).

**Table:** 

Title {1}	SHIFTPLAN: a randomized controlled trial investigating the effects of a multimodal shift-work intervention on drivers’ fatigue, sleep, health, and performance parameters
Trial registration {2a and 2b}. Trial registration on ClinicalTrials.gov	EDGE N°: 000339 NCT05452096
Protocol version {3}	July 18th, 2022_V14
Funding {4}	The budget has as yet not been approved by the public transport company. The current status of this trial therefore is “unfunded”.
Author details {5a}	• Corresponding author and Principal Investigator (PI): Inge Declercq, MD, neurologist, sleep expert. Multidisciplinary Sleep Disorders Center. Antwerp University Hospital (UZA). Drie Eikenstraat 655 - 2650 Edegem, Belgium. Email: inge.declercq@uza.be. In Section 31b (SPIRIT Guidance) referred to as ID. • Filip Van Den Eede, MD, PhD, Medical Coordinator, Department of Psychiatry, Antwerp University Hospital (UZA). Drie Eikenstraat 655 - 2650 Edegem, Belgium. Collaborative Antwerp Psychiatric Research Institute (CAPRI), Faculty of Medicine and Health Sciences, University of Antwerp (UA), Universiteitsplein 1, Antwerp 2610, Belgium. Email: filip.vandeneede@uza.be. In Section 31b (SPIRIT Guidance) referred to as FDVE. • Ella Roelant, PhD: Clinical Trial Center (CTC), CRC Antwerp, Antwerp University Hospital (UZA), University of Antwerp, Edegem, Belgium. Email: Ella.roelant@uza.be. In Section 31b (SPIRIT Guidance) referred to as ER. • Johan Verbraecken, MD, PhD, Medical Coordinator Multidisciplinary Sleep Disorders Center (UZA), LEMP FGGW UA. Antwerp University Hospital (UZA). Drie Eikenstraat 655 - 2650 Edegem, Belgium. Email: Johan.Verbraecken@uza.be. In Section 31b (SPIRIT Guidance) referred to as JV.
Name and contact information for the trial sponsor {5b}	Contact information: Inge Declercq, MD, neurologist, sleep expert. Multidisciplinary Sleep Disorders Center. Antwerp University Hospital (UZA). Drie Eikenstraat 655 - 2650 Edegem, Belgium. Email: inge.declercq@uza.be Trial sponsor: UNIVERSITY HOSPITAL ANTWERP, having its offices at Drie Eikenstraat 655, 2650 Edegem, legal entity number 0874.619.603, hereinafter referred to as “UZA”
Role of sponsor {5c}	Three collaborative partners will be involved: UZA, having its offices at Drie Eikenstraat 655, 2650 Edegem, legal entity number 0874.619.603, trial sponsor; ultimate authority over data management and collection, statistical analysis, monitoring, interpretation, writing of report, and publication. Public transport company: funding; provision of study participants. Occupational health service: intake and physical examination of eligible study participants and informed consent collection. UZA will review and analyze the pseudonymized personal data collected by the occupational health service from 176 drivers of the public transport company for the purposes of this research.

## Introduction

### Background and rationale {6a}

It is generally accepted that shift work is typically associated with disturbed life rhythms resulting from chronic exposure to circadian misalignment and sleep restriction, with long-term participation in most shift schedules causing serious health problems. Excessive fatigue and insomnia, for instance, are far more common among shift workers than they are in day workers and can lead to adverse effects such as reduced work performance, processing errors, accidents at work, absenteeism, reduced quality of life, anxiety, and depression [1, 2]. About one in three shift workers is affected by insomnia and up to 90% report regular fatigue and/or sleepiness at the workplace [3]. Moreover, epidemiological data shows that shift workers are at increased risk of cardiovascular disease, breast cancer, and shift work disorder. Prevalence estimates of shift-work disorder vary between 5 and 26.5% [3, 4]. The results of several epidemiological studies controlling for important potential confounders additionally suggest that exposure to shift work is an independent predictor of increased body weight [5] and body mass index (BMI) [6] and a higher prevalence of obesity [7]. For a comprehensive account of the short- and long-term health consequences of sleep disruption and circadian misalignment due to shift work, we refer to the excellent reviews of James and colleagues published in 2017 [8] and of Moreno and colleagues published in 2019 [9].

Given these widespread and serious health and functional consequences of shift work, there is a necessity for treatments that improve shift workers’ sleep quality and daily life and work performance. Most non-pharmacological recommendations to reduce insomnia and fatigue and enhance sleep quality mention improved scheduling, bright-light exposure, napping, psychoeducation fostering sleep hygiene, and cognitive-behavioral interventions [10, 11].

We performed computerized literature searches in PubMed using the following key terms: shift work disorder, fatigue, insomnia, shift work, drivers, measures, chronotype, circadian, treatment, intervention, strategies, and coping. The search was limited to non-pharmacological, human studies conducted and published as English language articles in peer-reviewed journals since 1999.

The search showed that the effects of shift work on the health, fatigue, and sleepiness of drivers have been robustly investigated in observational studies [12], as well as the effects of single measures such as scheduling or resting times [13], but that studies on the effectiveness of countermeasures against the adverse impact of shift work are sparse, especially for high-risk populations such as professional drivers. One study evaluating the effect of a stand-alone alertness management training program on sleepiness in long-haul truck drivers failed to provide evidence of its effectiveness [14]. Moreover, in their literature review, Sallinen et al. revealed that for all categories of shift systems, there was a lack of controlled intervention studies, hampering the development of solution-focused recommendations for shift scheduling [15]. Wong et al. [16] recently saw their Working Time Society consensus statements published, in which they expounded the need for a multi-level approach to managing occupational sleep-related fatigue, while in their also recent review of workplace interventions to promote sleep and health, Redeker et al. convincingly concluded that interventions using a single approach are unlikely to be effective. Highlighting the high public health burden associated with lack of recuperative sleep, the authors pointed out the pressing need to develop policies and implement programs aimed at improving workers’ sleep health [17, 18].

With SHIFTPLAN, we aim to fill this gap with comprehensive approaches. To our knowledge, we are the first to systematically gauge the effect of this dedicated, newly developed multimodal program that includes ergonomic shift scheduling and an educational program on well-defined health, sleep, and performance outcomes in professional drivers.

### Objectives {7}

#### Primary objective

To evaluate whether a 6-month implementation of a multimodal shift-work management program will help ameliorate the symptomatic burden (i.e., improve sleepiness indices) in professional public transport drivers working shifts.

We have opted for sleepiness as the primary outcome for the following reasons. Firstly, the criteria for Shift work disorder as defined by the International Classification of Sleep Disorders (ICDS-3) [19, 20] require the presence of sleepiness and/or insomnia. Secondly, sleepiness has been shown to be highly responsible for work-related and driving accidents [19, 21].

#### Key secondary objective

To examine whether the intervention has an effect on fatigue indices of the drivers.

It is to be noted that fatigue and sleepiness are two distinct states of being that may be present or absent independently from each other, where it is common to be fatigued without being sleepy and vice versa.

#### Supplementary secondary objectives

To examine whether the intervention has an effect on the following additional parameters:

- Health-related quality of life (HRQoL)
- Absenteeism
- Sleep outcomes
- Clinical health outcomes
- Mood and anxiety

At the conclusion of the study, we will conduct a qualitative assessment in terms of a subjective evaluation of the study among the study participants.

#### Exploratory objectives

The collected data will also be used to evaluate the outcomes as a function of the participants’ chronotype.

### Trial design {8}

Study design: This study is a randomized controlled interventional trial. It can be situated in occupational health and field research. The drivers will be 1:1 randomized to either the intervention or the control group according to a parallel group design. The study shall cover a period of 6 months.

The intervention is multi-modal and consists of three components: (a) healthy scheduling taking into account shift-rotation direction and speed, chronotype, resting time, and napping; (b) an education program specifically developed for shift workers; and (c) a dedicated information campaign for shift planners.

For a detailed description of the intervention, we refer to the “Intervention description **{**11a**}**” section and to the “Schematic overview of study progress” in supplementary material.

## Methods: participants, interventions, and outcomes

### Study setting {9}

Research population: Drivers of a public transport company, who work in multiple irregular shifts, often switching irregularly between late and early shifts with short recovery times.

The public transport company supplies public bus and tram services throughout multiple Belgian regions, employing at least 5500 drivers at the time of writing. Of this workforce, at least one-third is obese and about the same proportion has high blood pressure. Based on reports of the drivers themselves, 4% suffer from mental-health issues, alone leading to 49 sick leave days per year per employee.

Study setting: Data will be collected in two urban, geographically different regions that are similar with regard to work and stress load (intensity of traffic, mean number of passengers, variable and backward-rotating schedules with intermittent, restricted resting times).

### Eligibility criteria {10}

Inclusion criteria:

- Professional drivers having worked in shifts with the transport company in full-time or ≥ 80% part-time employment in the company’s regular backward-rotating schedule for at least 2 years.

Exclusion criteria:

- Regular medication for high blood pressure and uncontrolled high blood pressure (defined as exceeding 140/90 mmHg) at screening, regular medication for diabetes, sleeping pills, or sedative medication for depression (defined as trazodone, mirtazapine, and amitriptyline). Because our secondary outcomes imply the evolution of blood pressure and blood sugar parameters, drivers with such pre-existing controlled or non-controlled comorbidity will not be eligible for participation.
- High risk of moderate-to-severe obstructive sleep apnea syndrome (OSAS) as assessed with the STOP-Bang questionnaire, a simple, easy to remember, and self-reportable screening tool. We will use a cutoff score of 6 or higher to indicate the presence of OSAS [22].
- Drivers combining their job as a professional driver with another job elsewhere.
- Excessive sleepiness as defined as a score in excess of 12 on the Epworth Sleepiness Scale (ESS). Although the habitual cutoff is > 9, we opted for this higher threshold because we will be examining the effect of the intervention on daytime sleepiness. All applicants with an ESS > 12 will be excluded and referred to a general practitioner for further evaluation.
- A BMI higher than 35 kg/m^2^. We chose this cutoff value based on the data provided by the external occupational health service, which showed that in 2018, 42.6% of their drivers had a mean BMI of 25–30 and 27.5% had a BMI between 30 and 40.
- The presence of major depression as defined by a score exceeding the threshold of 1.75 on the Hopkins Symptom Checklist (HSCL-25), where higher scores were demonstrated to be highly indicative of depressive disorder according to the DSM-5 [23] and characterized as “a case requiring treatment.”

Quality assurance: The principal investigator (PI) and co-investigators are all certified medical specialists who will adhere to ICH-GCP guidelines to guarantee the quality of the research.

Statistics will be provided by qualified statisticians.

The PI is a neurologist and sleep expert who has a particular interest in and expert knowledge on circadian biology and shift-work management.

The co-investigators have extensive experience in the research of fatigue and/or sleep-wake disorders.

### Who will take informed consent? {26a}

Eligible applicants will be screened by occupational health physicians (OHPs) of the participating service regions who will explain all aspects of the study and check the in- and exclusion criteria during a pre-inclusion evaluation. When they consider the candidate eligible for inclusion and upon his/her agreement, the driver will be provided with written information and the consent form, which (s)he is asked to sign and return within a week of the assessment.

### Additional consent provisions for collection and use of participant data and biological specimens {26b}

Not applicable.

### Interventions

#### Explanation for the choice of comparators {6b}

The study will include an intervention and a control group.

The intervention provided to the intervention group (see also the “Intervention description {11a}” section) is based on an evidence-based good standard of care and includes:

- Healthy scheduling (fast forward-rotating shift schedules adapted to chronotype, adequate resting times, napping, bright-light therapy)
- Education program for drivers (psychoeducation promoting sleep hygiene, cognitive-behavioral strategies, stress-management techniques, information on chronotherapy such as bright-light therapy and napping)

The control group or “waiting-list group” will include drivers who will:

- Continue working according to the default shift schedules while being assigned to a waiting list in anticipation of the education program.

#### Intervention description {11a}

The duration of the intervention will be 6 months. This is a relatively liberal timeframe given that several intervention studies have shown that positive outcomes can be achieved between two weeks to 3 months, while educational programs as short as 1 to 3.5 h have been found to already exert a positive effect on sleep duration, alertness, and fatigue [24, 25]. However, given the complexity of factors involved in and affected by shift work, it is unlikely that in a real-life setting meaningful and consistent health benefits can be obtained by implementing a single or a limited number of interventions. As has been suggested by various experts in the field, this takes a holistic approach. We have explicitly chosen to test a multimodal intervention that addresses core issues, in line with Wong et al.’s [16] 2019 Working Time Society consensus statements on the need for a multi-level approach to managing occupational sleep-related fatigue. In their excellent review of workplace interventions to promote sleep and health also published in 2019, Redeker et al. stated that interventions using a single approach will not be effective and point to the pressing need to develop and implement policies to improve worker sleep health and the important public health problem associated with lack of recuperative sleep [17]. Several studies have addressed sleep training programs and their effectiveness on sleep parameters [24–26]. For a shift-work population, circadian challenges, limited time available for sleep, stress load, the effect of physical activity, and appropriate exposure to light on sleep-wake patterns, all need to be addressed. Psychoeducational programs should moreover include components based on cognitive-behavioral therapies for insomnia. The educational part of our intervention is in line with these expert recommendations. Ergonomic or healthy shift scheduling itself implies multiple measures: adapting the direction and cycle times of rotations to chronotypes, allowing for adequate resting times between shifts, and providing the possibility to take short naps in case of daytime/worktime sleepiness. The explicit choice for a multimodal intervention to be tested as a whole is thus in line with the assertion of other experts that workplace interventions using a single approach will not be effective.

Nevertheless, an embedded process evaluation of what did and did not work would be helpful in interpreting the final results. Therefore, we will also add a qualitative evaluation by means of a small questionnaire provided to the intervention group at the final assessment, in which they can provide their subjective evaluation of what measure provided the most benefit and why, and what part of the intervention did not work according to them and why.

Multimodal intervention: description of the three components

1. Implementing healthy scheduling. This will imply:
  - The introduction of forward-rotating shift schedules [13, 27, 28] with
  - Schedules being adapted to chronotype [29–31],
  - Allowing for adequate resting times between shifts, in particular after a late/night shift [13, 32]
  - Allowing for adequate resting times (i.e., a minimum of 48 h) between series of shifts
  - Allowing for napping, in particular during the first two days of an early shift [33, 34]

The optimization of shift schedules has been found a promising method to reduce the overall negative health impact associated with shift work [27]. Healthy, or ergonomic, shift scheduling comprises multiple measures: the modification of shift rotations (forward and fast) adapted to the individual worker’s chronotype and providing suitable between-shift resting times as well as napping facilities in case of daytime sleepiness.

For the sake of clarity and to avoid interference from ongoing schedules, the intervention group will, as a whole, be separated from all fellow drivers.

- A backward rotation schedule was prospectively related to an increased need for recovery as compared to a forward rotation schedule [13, 27]. Fast forward-rotating schedules have generally shown to be most beneficial [28].
- Allowing for chronotype finds its relevance in the need to reduce circadian misalignment and social jetlag [29, 35]. It is evident that late(r) chronotypes will adapt more easily to afternoon and late shifts, while the inverse applies for early chronotypes [31]. For example, Juda et al. [29] concluded that when working morning shifts, later chronotypes experienced the highest sleep constraints resulting in more pronounced signs of social jetlag, shorter sleep durations, and reduced sleep quality.
- Furthermore, van de Ven et al. [30] showed that workers whose chronotype did not align with the shift being worked had shorter sleep durations, lower quality of sleep, and longer recovery times. To add to the evidence, we will be comparing chronotype “pairs,” with drivers having nocturnal (evening and late-night) chronotypes working the opposite schedule from those of peers with diurnal (early and extremely early) chronotypes. A healthy schedule would then be a fast forward-rotating pattern with early chronotypes working more early shifts (3–4) than late chronotypes, with the inverse being applied for late(r) chronotypes.
- Several studies have highlighted the relevance of the length of the recovery period in-between two successive shift series and the absence of quick returns [32]. We will hence adopt resting times of at least 48 h between the last shift of a series and the first of a new series of shifts.
- Sleep deprivation and the resulting daytime sleepiness affect shift workers most at the start of early shifts. The rationale to offer drivers in our study early-morning bright-light chronotherapy (using Luminette®) prior to each early shift is twofold. We first expect the therapy to make drivers more alert and energetic and less sleepy during their working day. In a study by Bragard et al., the self-reported incidence of daytime slumbering had significantly decreased and the participants’ general health perception and physical functioning significantly improved after one month of Luminette® [36]. Secondly, we anticipate the therapy will counteract sleep deprivation by fostering the drivers’ biological sleep-wake cycle, potentially aiding them to go to sleep earlier in the evening following an early shift [37]. Light is known to be the most powerful synchronizer of endogenous circadian rhythms, where exposure to bright light in the early morning and avoidance of (bright) light in the evening should produce a phase advance. Drivers in the intervention group on early shifts will have the opportunity to use Luminette® and will be educated on the use of the device as well as on the impact of light on the sleep-wake cycle and on, among other topical issues, the importance of avoiding light in the evenings when working early shifts.
- Taking a short nap (15–30 min) has been shown to be an efficient method to prevent daytime sleepiness and boost alertness after a bad, sleep-deprived night [33, 34, 38]. Hence, drivers will be given the opportunity to take one 15-min morning nap during day 1 and/or day 2 of their early shift.
- The public transport company has an intelligent planning tool. It offers a modular solution for planning and analysis, scheduling, operations, and customer information, where each organization can select modules according to their own needs.

2. Education program for drivers

Our newly developed program targeting shift-working drivers involves:

- Psychoeducation on the sleep-wake cycle, biorhythms, and chronotypes and chronotherapy
- Cognitive and behavioral strategies and information based on the principles of the cognitive-behavioral treatment of insomnia (CBT-i)
- Information on the impact of light on wakefulness and sleep (including the use of bright-light therapy)
- Information on napping and creating awareness of and how to recognize daytime sleepiness
- Introducing tools for stress reduction
- Information on the advantages of physical exercise on the quality of the sleep-wake cycle
- Information on healthy eating in the context of shiftwork

If we want to promote the health of people working shifts and reduce fatigue or sleepiness, it is crucial to educate them about the importance and essence of healthy sleep-wake habits and to provide them with practical tools to help them handle the effects of changing shifts on their biorhythms in a more effective, conducive way. Studies evaluating education and sleep training programs for various shift-working populations and their effectiveness in improving sleep parameters and wakefulness [24–26] conclude that the various circadian challenges, ways to make the most of the limited time available for sleep, reduction of the work-related stress load, and the benefits of physical activity and sufficient exposure to light all need to be addressed.

CBT-i has been validated and is widely recommended as the most efficient and effective first-line treatment of insomnia and insomnia symptoms. The treatment comprises education on sleep-wake hygiene, stimulus control, and relaxation techniques, which components will all be included in our education program.

Furthermore, participants will be informed about and instructed in the practical use of bright-light therapy, nutrition, exercise, and napping. As to nutrition, since the results of dietary surveys show that the timing of meals is negatively affected by shift work [39] and a meta-analysis found that the prevalence of high-energy snacking is increased among shift workers [40], our program will address both proper meal timing and healthy nutrition.

The proposed education program has been developed by the PI and is founded on evidence-based principles described in the literature. Besides being a neurologist and sleep expert, she has also taken post-academic courses in education and coaching, has a university degree in CBT-i, and has ample experience in the delivery of training programs and CBT-i. However, to avoid bias, the PI will not be directly involved in the education of the drivers. Instead, she will be providing an extensive train-the-trainer program for certified trainers employed by the transport company who will be delivering the program.

The education program will consist of a one-day, 8-h session. The drivers randomized to the intervention group will complete the program within the first month after the conclusion of the randomization process (6 group sessions with a maximum of 15 participants per group). The drivers randomized to the control group will be informed that they have been placed on a waiting list and will be offered information on the program at a later date.

3. Information campaign for planners and team coaches

The campaign will cover:

- Epidemiological data and information on shift-work syndrome
- Explanation of the rationale and characteristics of healthy scheduling
- Information on chronotypes and available chronotype questionnaires to determine the drivers’ individual chronotypes and optimal chronotype-specific scheduling
- Provision of written informed consent stating that the data collected will remain strictly confidential and will only be used to create personalized schedules.

#### Criteria for discontinuing or modifying allocated interventions {11b}

Allocated interventions will not be modified.

When asked for their informed consent, all participants will be informed orally and in writing about their right to discontinue their participation to the study at any time.

Safety considerations:

- Applicants who are excluded according to the STOP-Bang Score criterion (scores ≥ 6), will be informed at intake about the risk of existing OSAS by the OHP and referred to a general practitioner to evaluate the necessity of referral to a sleep-wake clinic.
- Applicants with an ESS score > 12 will be excluded from participation and referred to their general practitioner.
- At any time, participants will have the opportunity to obtain more information on their questionnaire results.
- In case his/her intermediate score on the Checklist Individual Strength (CIS-20), a validated self-administered questionnaire evaluating fatigue that participants will be completing at several timepoints during the study, shows a marked increase, the participant will be interviewed again and re-examined.
- In case at intake depression (score of > 1.75 on the HSCL-25) is suspected, the driver will not be allowed to enter the study and referred to his/her general practitioner.

#### Strategies to improve adherence to interventions {11c}

Randomization will anticipate motivational bias, with candidate participants completing a short qualitative questionnaire to assess relevant motivational determinants.

Whether applicants eventually do or do not take part in the study will have no consequence whatsoever for any treatment they may need from the UZA or for their employment status.

Applicants and participants will be given a week to complete the various questionnaires.

The informed consent form will include a paragraph urging participants to follow all instructions as strictly as possible throughout the study period. To promote adherence to the daily sleep-wake diary, they will receive an email with a link to the sleep-wake diary every morning at 4 am to enable participants working an early shift to complete the diary before starting work. The public transport company will provide us with the drivers’ professional email addresses for this and other purposes.

By opting for a waiting list in the control condition we also aim to anticipate compensatory rivalry (where the control group could improve more than the intervention group). The control group will hence be informed that if the intervention proves effective, they will also be invited to complete the program in a second timeframe.

The intervention group will be separated from all other drivers for purposes of clarity and to avoid existing schedules from interfering with the experimental schedules.

If participants (still) have questions after completing the questionnaires, they will be given the opportunity to contact their occupational health physician by email and will be referred for appropriate professional help if indicated.

#### Minimizing contamination

Contamination will be very difficult, if not impossible, to avoid completely. Nevertheless, we reckon that contamination will remain restricted and at best provide better awareness of health habits in the control group. Since we, like other experts in the field, hold that workplace interventions aimed at optimizing shift schedules will not be effective using a single approach, our intervention will include healthy schedules and psychoeducation to address multiple factors such as individual variability, circadian challenges, time available for sleep, sleep hygiene, and stress load. The drivers in the control group will have no personal experience with or gain in-depth knowledge about any of these themes. For this reason, we do not expect that improvements in some health habits in the controls will lead to significant confounding changes. Moreover, the ergonomic schedules will be new to the company and specific for the drivers in the intervention group, which will be treated as a distinct group throughout the study period.

However, to control for contamination, we will add a question to each of the assessments to probe for any modifications in health habits during the study period. The investigators responsible for these assessments will be informed about the possibility of contamination as to raise their awareness on this issue.

#### Relevant concomitant care permitted or prohibited during the trial {11d}

The company has a stringent preventive and repressive policy on substance use (illicit drugs, alcohol, sedative medication) before and during working hours as part of the company’s legal obligation to prevent harm and protect the safety of its employees, passengers, and other road users.

None of the drivers randomized to the control group is allowed to join the educational program provided to the intervention group for the duration of the trial.

#### Provisions for post-trial care {30}

Not applicable.

### Outcomes {12}

#### Patient characteristics and baseline comparisons

Demographic and other baseline characteristics will be summarized per study group. For categorical variables, frequencies and percentages will be reported. Continuous variables will be summarized as means with standard deviations or medians with interquartile ranges as appropriate.

The following screening and baseline data will be collected:

- Age
- Gender
- Education
- Years of shift work
- BMI
- Smoking status (Y/N)
- Alcohol consumption
- Caffeine consumption
- Living and family status
- Physical activity
- Chronotype
- History of diabetes (on screening)
- History of depression (on screening)

#### Primary and key secondary endpoints: symptomatic burden (sleepiness, fatigue)

- Change in sleepiness from baseline to 6 months: sleepiness as assessed with the Epworth Sleepiness Scale (ESS).
- Change in fatigue from baseline to 6 months as assessed with the CIS (Checklist Individual Strength).

#### Outcome measurements regarding primary and key secondary endpoints (symptomatic burden)

- Daytime sleepiness: evolution of ESS scores as assessed at baseline and monthly up to and including the final 6-month evaluation and the statistical relevance of improvement (first to last score).
- Fatigue: evolution of CIS scores from baseline to 3 and 6 months and the statistical relevance of improvement (first to last score).

#### Analysis of the primary and key secondary endpoints (see also SPIRIT guidance 20a)

- The primary and key secondary endpoints (baseline to 6-month change in sleepiness and fatigue) will be analyzed using an independent samples t-test in the intention-to-treat population comparing the intervention to the control group.

#### Supplementary secondary endpoints: significant improvement on

- General health-related quality of life (HR-QoL as assessed with the SF-36).
- Absenteeism: sick leave in terms of the number of days off work due to illness will be derived from official records of and provided by the company.
- Sleep outcomes: Total sleep time (TST) and sleep efficiency (SE) as derived from self-recorded sleep-wake patterns and scores on the Pittsburgh Sleep Quality Index (PSQI).
- Clinical health outcomes: blood pressure, BMI, fasting blood glucose, glycosylated hemoglobin (HbA1c), high-sensitive C-reactive protein (hsCRP).
- Mood and anxiety as gauged with the Hopkins Symptom Checklist (HSCL-25).

#### Outcome measurements regarding secondary endpoints

- HR-Qol (SF-36): evolution of total SF-36 scores from baseline to 3 and 6 months and the statistical relevance of first-to-last score improvement
- Absenteeism: formal data collected and provided by public transport companies
- Sleep outcomes: scores on the PSQI at baseline, 3 and 6 months and the statistical relevance of the baseline to 6-month change. Evolution of mean total sleep time (TST) and sleep efficiency as derived from self-recorded/self-reported sleep-wake patterns
- Clinical health outcomes: blood pressure, BMI, fasting blood glucose, hemoglobin A1c, hsCRP: evolution of each measure from baseline, to 3 and up to 6 months and the statistical relevance of the improvement (first-to-last change). Analysis of the most relevant improvement
- Mood parameters as assessed with the HSCL-25 at baseline and at 6 months

#### Analysis of the secondary endpoints (see also SPIRIT guidance 20a)

- To evaluate the sensitivity of the results of the primary and key secondary outcome analysis, linear regression will be used to model the change in fatigue and the change in sleepiness, both from baseline to 6 months, with group as a predictor and taking into account potential confounders (i.e., gender, age, years of shift work, diabetes, BMI and smoking status).
- We will also conduct a per-protocol analysis in which only the data of the drivers who have fully adhered to the protocol will be compared.
- To handle missing data, we will use a mixed model with multiple imputation by chained equations. The imputation procedure will include intervention, gender, age, years of shift work, diabetes, BMI and smoking status, and the available CIS and ESS measurements, respectively. This will generate 20 completed datasets that will be analyzed separately. The results will be pooled.
- As fatigue and sleepiness will be assessed at three time points (baseline, 3 and 6 months), we will also test a linear mixed model with participant as a random effect to model the parameters’ evolution over time. This model allows for the correction of confounders and a between-group difference estimation at the different time points.
- To compare the continuous 6-month outcomes (SF-36, PSQI, HSCL-25, absenteeism, clinical health outcomes) for the two groups, we will use an independent samples *t*-test or Mann-Whitney *U* test (whichever is appropriate). Additionally, to correct for confounders, we may fit a linear regression model.
- A linear mixed model will be used to test the continuous outcomes (sleep times, CIS) over time.
- The qualitative sleep-wake data will be collected and analyzed by a co-investigator at the Antwerp University Hospital.

#### Qualitative assessment of implementation and proximal effect of the intervention (change process) by short interview provided to both groups at the end of the study

Questions posed at the end of the study:

1. To what extent are you satisfied with your well-being? (To be rated on a 7-point Likert scale ranging from extremely satisfied to extremely dissatisfied)
2. If your experience was positive, can you explain why this is? Which part of the intervention seemed most useful to you and why?
3. If your experience was negative, can you explain why this is? Which part of the intervention seemed most useless to you and why?
4. Intervention group: Would you like to continue working your new schedule? Control group: Would you like to change to a new personalized schedule?

#### Exploratory analysis (see also SPIRIT guidance 20b)

- Since we are interested to see whether the intervention effects will differ depending on chronotype, we will be looking for interactions between the intervention and the various chronotypes.
- We will also be exploring associations between chronotype and the other variables tested (i.e., BMI, mental-health status (HSCL-25), fatigue, and sleepiness indices).

### Participant timeline {13}

See also “Schematic overview of study progress” (Supplementary material).

Besides keeping a daily online sleep-wake diary (with a link to the diary being provided daily by email), participants will be asked to participate in the following evaluations:

1. Screening session to test in- and exclusion criteria and obtain informed consent
2. Baseline—intake visit including all assessments/examinations and randomization
3. Full assessment 3 months after start of the program
4. Full assessment 6 months after the start of the program

Fatigue will be monitored on a more regular basis. Participants will be asked to fill out the CIS at baseline and once a month for the duration of the intervention (Table 1).

**Table 1:** Schematic study overview

	Visit to occupational health physician (at least 10 h prior to fasting!)	Clinical examination	Blood sample drawn (after a minimum of 10 h fasting)	Self-report questionnaire(s) (time needed)	Sleep-wake diary (time needed)
Appointment 1 (intake)	**✓**	**✓**	**✓**	**✓**(20 min)	
1× each month				**✓**Fatigue scale (5–10 min)	
Appointment 2 (after 3 months)	**✓**	**✓**	**✓**	**✓**(20 min)	
Appointment 3 (after 6 months)	**✓**	**✓**	**✓**	**✓**(25 min)	
Daily from trial start to end					**✓** (2–5 min)

This overview (translated from Dutch) will be included in the informed consent documentation

### Sample size {14}

For the primary and key secondary research question, we will be looking at two outcome measures, namely change in sleepiness (ESS) and in fatigue (CIS), both from baseline to the 6-month endpoint. We expect the outcomes for the intervention group to be superior to those obtained in the control group.

With regard to ESS, Patel et al. [41] mentioned 2 or 3 units as being indicative of a clinically minimally important difference in obstructive sleep apnea. Viitasalo et al. [13] found the largest SD for the ESS to be 5.3 units. Assuming a standard deviation of 5.3 and a significance level of 0.05, an achieved sample size of 72 per group is required to detect an effect of 2.5 with 80% power using a two-sample t-test. Taking into account at least 20% drop-out, 88 drivers will be recruited into each group. Hence, a total of 176 drivers will be recruited for the study.

As to the CIS, we will consider a drop of 10 units to reflect a clinically minimally important difference (CMID). This number is in part based on Vercoulen et al. [42, 43] who considered scores of 27 or higher to indicate abnormal fatigue and scores ≥ 35 severe fatigue, where a drop of 8 or more units signifies a change in severity classification. Additionally, we refer to Worm-Smeitink et al. [44] who, comparing CIS scores for breast cancer survivors and healthy controls in 2017, observed a 10-unit difference between the study population and the controls, with the former reporting higher levels of fatigue. We are thus confident that a difference of 10 units denotes a clinically relevant change.

For the standard deviation, we rely on Beurskens et al. [45] who reported an SD of 18.9 units for a blue-collar population. Assuming a standard deviation of 19 and a significance level of 0.05 an achieved sample size of 58 per group would be required to detect an effect of 10 with 80% power using a two-sample *t*-test. Taking into account at least 20% drop-out, 70 drivers should be recruited into each group.

Based on these findings, we will adopt a minimum of 70 for each group in our study. Taking into account a drop-out of 20%, we will be recruiting 88 drivers per group (i.e., 176 drivers overall) to achieve this. Hence, with a total recruitment of 176 drivers, the study is sufficiently powered for both primary and key secondary outcomes. A power of 80% was a compromise as we needed the recruitment to be feasible and still be able to achieve sufficient power.

### Recruitment {15}

In each of the two service regions of the company taking part in the study, recruitment will be via posters and information screens in the respective terminals and online via their intranet, inviting drivers working in the relevant regions to apply for participation in the study either by mail, email or telephone. All the drivers in the relevant service regions will also receive an individual invitation by email via their professional email account provided by the company for internal communications.

### Assignment of interventions: allocation

#### Sequence generation {16a}

Stratified randomization will be used to allocate the drivers 1:1 to the intervention or control group. Stratification will be by service region (*n* = 2), age (younger or older than 40 years), gender and BMI (BMI ≤ 25; BMI 26–30; BMI 31–35). We will employ Qminim, a web-based randomization system that uses minimization to ensure a similar distribution of the stratifying factors between the study conditions.

#### Concealment mechanism {16b}

After the intake visit with the occupational health physician (OHP) of the relevant service region, all data of the drivers that have agreed to participate and have given their informed consent will be coded, and therefore, encrypted data will be sent to the PI for further evaluation.

The stratified randomization will be done by Qminim a web-based randomization system which uses minimization to ensure a similar distribution of the stratifying factors between the study conditions.

The administrator of the coded database will be one of the OHPs. The coded data will be stored and accessible for at least 20 years, with the encryption key remaining with the occupational health service. All investigators will only have access to the coded data.

#### Implementation {16c}

See also above: 16b

The OHPs will be responsible for the enrolment of the drivers and the coding of service regions (*n* = 2), age (≤ than 40 years vs. > 40 years), gender, and BMI (≤ 25; 26–30; 31–35). Since the participants randomized to the intervention group will be assigned to a rotation schedule adapted to their chronotype (three types), codes for the chronotypes will be created. The Qminim system will assure anonymized and random assignment of the participants to the two conditions.

### Assignment of interventions: blinding

#### Who will be blinded {17a}

The statistical analyst and the PI will be blinded to the participants’ identity and only have access to coded data.

#### Procedure for unblinding if needed {17b}

Not applicable.

### Data collection and management

#### Plans for assessment and collection of outcomes {18a}

All (eligible) participants will be examined by an occupational health physician prior to the study (screening visit), at intake (baseline) and at two scheduled time points (3 and 6 months).

See also “Schematic overview of study progress” (Supplementary material, Timeline), which also describes which data will be collected when.

- The occupational health physicians (OHP) will be collecting the following socio-demographic data at intake (baseline): date of birth, gender, living and family status, education, years of shift work, smoking status, alcohol and caffeine consumption, and physical activity.
- An OHP and trained nurse (blood draw) will be collecting the following clinical health data: blood pressure, BMI, fasting plasma glucose, glycosylated hemoglobin (HbA1c), hsCRP. Fasting plasma glucose and HbA1c will be taken after a fasting period of 10 h. All clinical visits will be scheduled on day shifts between 8 and 10 am.
- The following online self-report questionnaires will be used (in validated translation): the Morningness-Eveningness Questionnaire (MEQ), the Pittsburgh Sleep Quality Index (PSQI), the Epworth sleepiness scale (ESS), the Checklist Individual Strength (CIS), the Medical Outcomes Short Form 36 Health Status Survey (SF-36), the STOP-Bang questionnaire, and the Hopkins Symptom Checklist (HSCL-25)
- Online sleep-wake diaries will be used to collect qualitative self-reported data on sleep-wake habits and sleep-wake patterns, mean total sleep time (TST), and sleep efficiency (SE) on a daily basis.

Evaluations:

1. Screening for in- and exclusion criteria and informed consent
2. Baseline—intake visit and randomization
3. Outcomes 3 months after the start of the program
4. Outcomes 6 months after the start of the program

Fatigue will be monitored on a more regular basis. Participants will be asked to fill out the CIS at baseline and subsequently once a month throughout the intervention.

**Explication and rationale of proposed questionnaires and clinical health measurements**

*STOP-Bang questionnaire* (Chung et al., 2008) [46]:

The STOP-Bang questionnaire is a simple, user-friendly self-report screening tool that evaluates four subjective items (STOP: Snoring, Tiredness, Observed apnea and high blood Pressure) and four demographics items (Bang: BMI, age, neck circumference, gender) and has been validated for assessing OSAS. In their meta-analysis, Nagappa et al. [22] demonstrated its sensitivity to be 90%, 94%, and 96% in detecting any OSAS (AHI ≥ 5), moderate-to-severe OSAS (AHI ≥ 15), and severe OSAS (AHI ≥ 30), respectively. In the sleep clinic population, the probability of severe OSAS with a score of 3 was 25%. With a stepwise increase of the score to 4, 5, 6, and 7/8, the probability rose proportionally to 35%, 45%, 55%, and 75%, respectively. Given that with scores of 6 or higher the risk probability for moderate-to-severe OSAS was 55% [22] and moderate-to-severe OSAS could bias the reasons for being sleepy in our study, we will use a cut-off score of ≥ 6 to exclude drivers from participating.

*Morningness-Eveningness Questionnaire* (Horne and Ostberg) [47]:

This self-assessment scale is used to determine morningness-eveningness in human circadian rhythms and has 19 items subdivided into subscales. MEQ scores are to be added and can vary from 16 to 86. Scores lower or equal to 41 typify respondents as an “evening type,” scores exceeding 59 as a “morning type,” and scores between 42 and 58 as a “neutral type,” which categories we will be using to determine the chronotype of our drivers to enable us to adapt shift schedules to their types as much as possible to thus try and reduce any circadian misalignment and social jet lag.

*Checklist Individual Strength* (CIS-20): [42, 43]:

The CIS is a validated, self-administered questionnaire assessing fatigue. It consists of 20 statements for which the respondent has to indicate on a 7-point Likert scale to what extent the particular statement applies to him or her and takes 5–10 min to complete. The statements refer to aspects of fatigue experienced during the previous 2 weeks. Four dimensions are gauged: the subjective experience of fatigue and potential reductions in motivation, activity, and concentration. The final score is obtained by adding the scores to all questions (range 20–140), where scores of 27 or higher are taken to indicate abnormal fatigue and scores ≥ 35 severe fatigue. Scores in excess of 76 have been associated with a high risk of chronic absenteeism in a working population [48].

The internal consistency of the CIS was shown to be good: Cronbach’s alpha for the total CIS was 0.90 and for the subscales the alphas ranged from 0.83 to 0.92. The CIS was found to discriminate between individuals with chronic fatigue syndrome, with multiple sclerosis and healthy controls and the convergent validity was also satisfying. The CIS has been shown to be an appropriate instrument for assessing fatigue in working populations [45].

*Epworth Sleepiness Scale* (ESS) [49]:

This validated self-report scale gauges daytime sleepiness. Many studies have shown daytime sleepiness and drowsy driving to be major risk factors for road accidents and reduced performance and a serious issue in shift workers [12]. The eight items of the ESS ask the respondent how likely (s)he is to doze off or fall asleep in different situations of everyday life including (1) sitting and reading, (2) watching TV, (3) sitting (inactive) in a public place (theater, meeting), (4) as a passenger in a car for 1 h or longer, (5) when lying down to rest in the afternoon when circumstances permit, (6) when sitting and talking to someone, (7) when sitting quietly after lunch without alcohol, and (8) in a car while stopping in traffic for a few minutes. The respondents can rate the chance of dozing for each item as never (score = 0), slight (score = 1), moderate (score = 2), or high (score = 3). Total scores can range from 0 to 24 and ESS scores exceeding 9 are considered indicative of daytime sleepiness. The ESS takes 5 min to be completed.

*The Medical Outcomes Short Form 36 Health Status Survey* (SF-36) (Aaronson et al. 1998) [50]:

The SF-36 [50] is one of the most widely used generic self-report measures of health-related quality of life and consists of 36 items that are structured into nine subscales: physical functioning (10 items), social functioning (2 items), role functioning-physical (4 items), role functioning-emotional (3 items), mental health (5 items), vitality (4 items), body pain (2 items), general health (5 items), and reported health transition (1 item). The questionnaire generates two summary scores: the physical component summary and the mental component summary. The scales are scored from 0–100 (transformed scale = (actual raw score − lowest possible raw score)/possible raw score range) × 100), with higher scores indicating better health. The measure has been demonstrated to have strong internal consistency (*α* ≈ 0.80), validity, and reliability [51].

*Pittsburgh sleep quality index (PSQI)* (Buysse et al., 1989) [52]:

The PSQI is a self-rated questionnaire that assesses sleep quality and disturbances over a 1-month interval. Nineteen individual items generate seven “component” scores: subjective sleep quality, sleep latency, sleep duration, habitual sleep efficiency, sleep disturbances, use of sleeping medication, and daytime dysfunction. The sum of scores for these seven components yields one global score. For sleep health to be more objectively measured, we would have to use polysomnography. This is, however, not feasible within the framework of our field study. In order to sufficiently power the trial, we will be following two large groups and cannot expect all our participants to travel to and from the sleep center on multiple occasions since this would have to be done on off-duty days. Moreover, polysomnography also has its limitations as a sleep-health measure due to first-night effects. We have thus opted for the Pittsburgh Sleep Quality Index. Although self-rated, the PSQI is widely used to assess sleep in sleep and shift-work research, where a global PSQI score > 5 was shown to yield a diagnostic sensitivity of 89.6% and specificity of 86.5% (kappa = 0.75, *p* ⩽0.001) in distinguishing good and poor sleepers. Acceptable measures of internal homogeneity, consistency (test-retest reliability), and validity were obtained. The clinimetric and clinical properties of the PSQI thus suggest its utility both in clinical practice and (field) research.

*The Hopkins Symptom Checklist - 25 (HSCL-25)* (Parloff et al., 1954) [53–56]:

This self-administrated questionnaire helps to assess and measure anxiety and depression in multiple settings. Anxiety and depression show considerable overlap in primary-care populations, and its brevity and self-report format make the scale well-suited for use in the busy primary-care setting. The original US version has been shown valid, reliable, and ergonomic. We will be using the Dutch version, translated and validated by Kleijn et al [57]. Translations have been shown to be adequate and applicable for multiple cultures. Similar to clinical interviews, its specificity is robust, being between 0.78 to 0.88, with its reliability (Cronbach’s alpha) ranging from 0.87 to 0.97. The scale’s 25 questions are divided into two subsections relating to the presence and intensity of symptoms of depression and anxiety as experienced during the previous week. Respondents rate the items on a 4-point Likert scale, ranging from 1 (strongly disagree) to 4 (completely agree), which takes between five to 10 min. The final score ranges from 1.00 to 4.00 and is calculated by dividing the sum of the scores of all items by 25. Generally, scores over 1.75 are taken to be indicative of a major depression and defined as “a case requiring treatment.” This cutoff of 1.75 showed a sensitivity of 73%, a specificity of 76%, a positive predictive value (PPV) of 58%, and a negative predictive value (NPV) of 86%.

### Use and choice of clinical health parameters

The clinical health parameters that we will be collecting (i.e., blood pressure in mmHg, BMI in kg/m^2^, fasting plasma glucose in mmol/l, glycosylated hemoglobin in mmol/mol (HbA1c), hsCRP ) have been shown to be related to cardiovascular health. Shiftwork is associated with a higher risk of cardiovascular disease [58, 59] and has also been shown to be an independent risk factor for the development of hypertension. Moreover, high blood pressure is positively related to mortality from cerebrovascular disease, with the prevalence of ischemic stroke being higher in shift workers than in the general population [13]*.* Puttonen et al [60] have shown that hsCRP levels were higher among shift workers in models adjusted for age and recent infectious diseases. Also, raised fasting blood glucose and glycosylated hemoglobin, as well as a high BMI, are known risk factors for diabetes and cardiovascular disease. Given that shift work is associated with each of these three risk factors, it seems plausible to suggest that these health threats are at least partially attributable to the circadian misalignment many shift workers experience, which situation we are attempting to ameliorate by the introduction of healthy scheduling and psychoeducation.

Normal fasting blood glucose concentration according to the WHO is a value between 3.9 and 5.6 mmol/l. On average, normal HbA1c for non-diabetics is < 36 mmol/mol. Normal BMI is defined as a value lower than 25 kg/m^2^. Normal blood pressure is defined as a value lower than 140/90 mmHg. General guidelines for hsCRP scores: low risk of cardiovascular disease, less than 1.0 mg/L; average risk, 1.0 to 3.0 mg/L; and high risk, above 3.0 mg/L.

Absenteeism, defined as the number of sick leave days, will be derived from data recorded and provided by public transport company.

#### Plans to promote participant retention and complete follow-up {18b}

Considering that completion of the questionnaires can take up to 40 min, participants will be allowed a week to return the completed scales.

The informed consent form will include a paragraph urging participants to follow all instructions as strictly as possible throughout the study period. To promote adherence to the daily sleep-wake diary, they will receive an email with a link to the sleep-wake diary every morning at 4 am to enable participants working an early shift to complete the diary before starting work. The company will provide the data manager with the drivers’ professional email addresses for this and other purposes.

By opting for a waiting list in the control condition we also aim to anticipate compensatory rivalry (where the control group could improve more than the intervention group). The control group will hence be informed that if the intervention proves effective, they will also be invited to complete the program in a second timeframe.

The intervention group will be separated from all other drivers for purposes of clarity and to avoid existing schedules from interfering with the experimental schedules.

If participants (still) have questions after completing the questionnaires, they will be given the opportunity to contact their occupational health physician by email and will be referred for appropriate professional help if indicated.

### Data management {19}

All the driver-participants will receive the links to the online questionnaires and daily sleep-wake diary by email addressed to their professional accounts provided by the public transport company to all their employees in 2019. We will be employing electronic case report forms (eCRF) using Research Electronic Data Capture (REDCap) platform software, a secure data collection tool that meets HIPAA compliance standards (https://www.project-redcap.org/).

Only coded data encrypted by the occupational health physicians will be used for analysis. All data will be stored and remain accessible for at least 20 years, with the encryption key being known to participating occupational health physicians only.

Kim Claes will be responsible for the development of the eCRF. Kim Claes is an IT expert experienced in eCRF who is affiliated with both the UZA and University of Antwerp’s Clinical Trial Center (Edegem campus). Email: datamanagement@uza.be

### Confidentiality {27}

All procedures will be GDPR (General Data Protection Regulation) in accordance with procedures stipulated by UZA’s Clinical Trial Center (CTC).

Privacy and confidentiality are further safeguarded by a non-disclosure agreement and the use of anonymized, coded data that will remain encrypted for the companies’ human resource department and other management levels to ensure that participation in the study will have no effect whatsoever on the participants’ employment status.

### Plans for collection, laboratory evaluation, and storage of biological specimens for genetic or molecular analysis in this trial/future use {33}

A total of three blood samples will be drawn from all study participants: the first at intake, the second after 3 months of starting the program, and the third at the 6-month endpoint. Serum samples will be analyzed for hsCRP, glycosylated hemoglobin, and fasting plasma glucose. If a participant has not respected the 10-h fasting period preceding a draw, another visit with the trained nurse will be scheduled within a week to collect a correct sample. The samples will be stored for 20 years.

## Statistical methods

### Statistical methods for primary and secondary outcomes {20a}

See also Statistical Analysis Plan (SAP) in the supplementary file

### Analysis of the primary and key secondary endpoints

The primary endpoint and key secondary outcomes (changes in sleepiness and in fatigue) will be analyzed using an independent samples *t*-test in the intention-to-treat population comparing the intervention to the control group. Besides this, a linear regression model will be used to model the change in fatigue and the change in sleepiness from baseline to 6 months, with group as a predictor and taking into account possible confounders gender, age, years of shift work, diabetes, BMI, and smoking status [61].

### Analysis of the supplementary secondary endpoints

- To evaluate the sensitivity of the results of the primary outcome analysis, linear regression will be used to model the changes in fatigue and sleepiness from baseline to 6 months, with group as a predictor and taking into account potential confounders, i.e., gender, age, years of shift work, diabetes, BMI, and smoking.
- We will also be running a per-protocol analysis in which only the data of drivers who have completed the full protocol will be compared.
- As the fatigue and sleepiness are measured at baseline, 3 and 6 months we will also consider a linear mixed model with subject as a random effect to model their evolution over time. This model allows for the correction of confounders and the difference between the groups can be estimated at different time points.
- To handle missing data, we will use a mixed model with multiple imputation by chained equations. The imputation procedure will include intervention, gender, age, years of shift work, diabetes, BMI and smoking status, and the available CIS and ESS measurements, respectively. This will generate 20 completed datasets that will be analyzed separately. The results will be pooled.
- To compare the continuous outcomes (SF-36, PSQI, HSCL-25, absenteeism, clinical health outcomes) at 6 months between the two groups, we will use an independent samples *t*-test or Mann-Whitney *U* test (whatever is appropriate). We can also fit a linear regression model for these outcomes which makes it possible to correct for confounders.
- A linear mixed model will be studied for the continuous outcomes (sleep times, CIS) measured over time.

### Interim analyses {21b}

Not applicable.

### Methods for additional analyses (e.g., subgroup analyses) {20b}

#### Exploratory analysis

- We are interested to see if the intervention effect is different depending on the chronotype; hence, an interaction between intervention and chronotype will be considered.
- We are interested in the association between chronotype and other variables like BMI, mental health (as tested by the HSCL-25), fatigue scores, and sleepiness scores; hence, this will also be explored.

### Methods in analysis to handle protocol non-adherence and any statistical methods to handle missing data {20c}

Handling of protocol adherence: primary analyses will be according to intention-to-treat principles; the controls randomized to the waiting-list condition will be analyzed the same way as those randomized to the intervention group, with the per-protocol population being the study participants who attended the education program and worked the new, chronotype-based shift for the full 6 months.

Handling of missing data: The proposed linear mixed model allows for missing values at certain time points as the method uses all available data points per participant and the missing at random assumption, implying that missing values are assumed to be dependent on the observed responses, which seems a reasonable premise in our case. In case of large proportions of missing values, we will employ multiple imputation techniques. The imputation procedure will include intervention, gender, age, years of shift work, diabetes, BMI and smoking, and the available CIS and ESS measurements respectively. Twenty completed datasets will be generated in this way and analyzed separately. The results will be pooled.

### Plans to give access to the full protocol, participant-level data, and statistical code {31c}

Not applicable.

### Oversight and monitoring

#### Composition of the coordinating center and trial steering committee {5d}

Data monitoring committee: Monitoring will be performed at the Clinical Trial Center (CTC) Antwerp and consist of:

- One on-site initiation visit (SIV) per location
- One close-out visit (COV) per location
- Two on-site monitoring visits per location during the 6-month study period

The CTC is also the coordinating center and will provide the PI with a written report of each visit.

A steering committee will be composed.

#### Composition of the data monitoring committee, its role, and reporting structure {21a}

See 5d.

#### Adverse event reporting and harms {22}

Not applicable to this trial. As no unknown or new medication, substances or procedures are involved in this trial, it is highly unlikely that the intervention will have any adverse side effects.

#### Frequency and plans for auditing trial conduct {23}

See 5d.

#### Plans for communicating important protocol amendments to relevant parties (e.g. trial participants, ethical committees) {25}

Any pertinent modification of the protocol will first be discussed within the Steering Committee and, if agreed to, communicated to the Ethics Committee for approval.

#### Dissemination plans {31a}

As PI, Inge Declercq will take the lead in all publications. No publication restrictions apply.

This research and the resulting findings will also be included in her doctoral dissertation.

## Discussion

Expecting favorable outcomes, we will be contributing to the amelioration and the (partial) prevention of the widespread and debilitating health and social consequences resulting from working shifts. Besides publications in peer-reviewed journals to disseminate the results of our trial, we also aim to provide evidence-based guidelines on the healthy management of shift work overall. Together, our findings and recommendations are to help reduce the high public-health and economic burdens associated with shift work.

### Trial status

Last version of protocol: V14- July 18, 2022.

Recruitment was originally planned to start in June 2020 but due to the COVID-19 pandemic, the trial has been put and still is on hold. Recruitment is now planned to start in Autumn 2022.

## Supplementary Information

**Additional file 1.**

**Additional file 2.**

**Additional file 3.**

## Data Availability

The PI, statistical analyst, and data manager will have access to the final trial dataset. Data of the participants will be collected in the context of this study only and will be owned by the current researchers. The data will be collected by physicians of external occupational health services of the company, which will participate as an independent partner. Its independence and the fact that the data collected will be anonymized and used exclusively for research purposes will be guaranteed contractually. A non-disclosure agreement will be signed by the occupational health physicians. The planners with the public transport company will be asked to sign a non-disclosure agreement to ensure the information on chronotype will remain strictly confidential and will only be used to develop the experimental shift schedules. The information regarding absenteeism will exclusively be used for the purpose of this study and will have no impact on the participants’ employment status whatsoever. The data will only be accessible for a limited number of people and will remain encrypted to the investigators.

## Abbreviations

BMI: Body mass index
HbA1c: Glycosylated hemoglobin
hsCRP: High-sensitive C-reactive protein
OSAS: Obstructive sleep apnea syndrome
STOP-Bang: Snoring, tiredness, observed apnea and high blood pressure and BMI, age, neck circumference, gender
MEQ: Morningness-Eveningness Questionnaire
CIS-20: Checklist individual strength
ESS: Epworth Sleepiness Scale
PSQI: Pittsburgh Sleep Quality Index
HSCL-25: Hopkins Symptom Checklist
SF-36: Medical Outcomes Short Form 36 Health Status Survey
CMID: Clinical minimally important difference
TST: Total sleep time
SE: Sleep efficiency
CBT-i: Cognitive-behavioral therapy for insomnia
SD: Standard deviation
PPV: Positive predictive value
NPV: Negative predictive value
OHP: Occupational health physician

## References

[1] Drake CL, Roehrs T, Richardson G, Walsh JK, Roth T (2004). Shift work sleep disorder: prevalence and consequences beyond that of symptomatic day workers. Sleep..

[2] Eldevik MF, Flo E, Moen BE, Pallesen S, Bjorvatn B. Insomnia, excessive sleepiness, excessive fatigue, anxiety, depression and shift work disorder in nurses having less than 11 hours in-between shifts. PLoS One. 2013;8(8):e70882. 10.1371/journal.pone.0070882. 10.1371/journal.pone.0070882 PMC3744484 23976964

[3] Richter K, Acker J, Adam S, Niklewski G (2016). Prevention of fatigue and insomnia in shift workers - a review of non-pharmacological measures. EMPA J.

[4] Pallesen S, Bjorvatn B, Waage S, Harris A, Sagoe D (2021). Prevalence of Shift Work Disorder: A Systematic Review and Meta-Analysis. Front Psychol.

[5] Geliebter A, Gluck ME, Tanowitz M, Aronoff NJ, Zammit GK (2000). Work-shift period and weight change. Nutrition..

[6] Parkes KR (2002). Shift work and age as interactive predictors of body mass index among offshore workers. Scand J Work Environ Health.

[7] Di Lorenzo L, De Pergola G, Zocchetti C, L’Abbate N, Basso A, Pannacciulli N, Cignarelli M, Giorgino R, Soleo L (2003). Effect of shift work on body mass index: results of a study performed in 319 glucose-tolerant men working in a Southern Italian industry. Int J Obes Relat Metab Disord.

[8] James SM, Kimberly AH, Gaddameedhi S, Van Dongen HPA (2017). Shift work: disrupted circadian rhythms and sleep-implications for health and well-being. Curr Sleep Med Rep.

[9] Moreno C, Marqueze EC, Sargent C, Wright KP, Ferguson SA, Tucker P (2019). Working Time Society consensus statements: Evidence-based effects of shift work on physical and mental health. Ind Health.

[10] Jehan S (2017). Shift work and sleep: medical implications and management. Sleep Med Disord.

[11] Wright KP, Bogan RK, Wyatt JK (2013). Shift work and the assessment and management of shift work disorder (SWD). Sleep Med Rev.

[12] Higgins JS, Michael J, Austin R, Äkerstedt T, Van Dongen HPA, Watson N, Czeisler C, Pack AI, Rosekind MR (2017). Asleep at the wheel-the road to addressing drowsy driving. Sleep..

[13] Viitasalo K, Kuosma E, Laitinen J, Härmä M (2008). Effects of shift rotation and the flexibility of a shift system on daytime alertness and cardiovascular risk factors. Scand J Work Environ Health.

[14] Pylkkönen M, Tolvanen A, Hublin C, Kaartinen J, Karhula K, Puttonen S, Sihvola M, Sallinen M (2018). Effects of alertness management training on sleepiness among long-haul truck drivers: a randomized controlled trial. Accid Anal Prev.

[15] Sallinen M, Kecklund G (2010). Shift work, sleep, and sleepiness – differences between shift schedules and systems. Scand J Work Environ Health.

[16] Wong IS, Popkin S, Folkard S (2019). Working Time Society consensus statements: a multi-level approach to managing occupational sleep-related fatigue. Ind Health.

[17] Redeker NS, Caruso CC, Hashmi SD, Mullington JM, Grandner M, Morgenthaler TI (2019). Workplace interventions to promote sleep health and an alert, healthy workforce. J Clin Sleep Med.

[18] Ritonja J, Aronson KJ, Matthews RW, Boivin DB, Kantermann T (2019). Working Time Society consensus statements: Individual differences in shift work tolerance and recommendations for research and practice. Ind Health.

[19] Gonçalves M, Amici R, Lucas R, Åkerstedt T, Cirignotta F, Horne J, Léger D, McNicholas WT, Partinen M, Téran-Santos PP, Grote L (2015). & National Representatives as Study Collaborators. Sleepiness at the wheel across Europe: a survey of 19 countries. J Sleep Res.

[20] American Academy of Sleep Medicine. The International Classification of Sleep Disorders – Third Edition (ICSD-3). Darien: American Academy of Sleep Medicine, 2014. ISBN: 0991543416. https://aasm.org.

[21] Uehli K, Mehta AJ, Miedinger D, Hug K, Schindler C, Holsboer-Trachsler E, Leuppi JD, Künzli N (2014). Sleep problems and work injuries: a systematic review and meta-analysis. Sleep Med Rev.

[22] Nagappa M, Liao P, Wong J, Auckley D, Ramachandran SK, Memtsoudis S, Mokhlesi B, Chung F (2015). Validation of the STOP-Bang Questionnaire as a screening tool for obstructive sleep apnea among different populations: a systematic review and meta-analysis. PLoS One.

[23] American Psychiatric Association. Diagnostic and statistical manual of mental disorders (5th ed.). 2013. https://doi-org.ezproxy.frederick.edu/10.1176/appi.books.9780890425596.

[24] Rosekind MR, Gregory KG, Mallis MM (2006). Alertness management in aviation operations: enhancing performance and sleep. Aviat Space Environ Med.

[25] Scott LD, Hofmeister N, Rogness N, Rogers AE (2010). Implementing a fatigue countermeasures program for nurses: a focus group analysis. J Nurs Adm.

[26] Chen PH, Kuo HY, Chueh KH (2010). Sleep hygiene education: efficacy on sleep quality in working women. J Nurs Res.

[27] van Amelsvoort L, Jansen N, Swaen G, van den Brandt PA, Kant I (2004). Direction of shift rotation among three-shift workers in relation to psychological health and work-family conflict. Scand J Work Environ Health.

[28] Neil-Sztramko SE, Pahwa M, Demers PA, Gotay CC (2014). Health-related interventions among night shift workers: a critical review of the literature. Scand J Work Environ Health.

[29] Juda M, Vetter C, Roenneberg T (2013). Chronotype modulates sleep duration, sleep quality, and social jet lag in shift-workers. J Biol Rhythms.

[30] van de Ven HA, van der Klink JJ, Vetter C, Roenneberg T, Gordijn M, Koolhaas W, de Looze MP, Brouwer S, Bültmann U (2016). Sleep and need for recovery in shift workers: do chronotype and age matter?. Ergonomics..

[31] Hittle BM, Gillespie GL (2018). Identifying shift worker chronotype: implications for health. Ind Health.

[32] Hakola T, Paukkonen M, Pohjonen T (2010). Less quick returns - greater well-being. Ind Health.

[33] Gillberg M, Kecklund G, Axelsson J, Akerstedt T (1996). The effects of a short daytime nap after restricted night sleep. Sleep..

[34] Horne JA, Reyner LA (1996). Counteracting driver sleepiness: effects of napping, caffeine, and placebo. Psychophysiology..

[35] Vetter C, Fischer D, Matera JL, Roenneberg. (2015). Aligning Work and Circadian Time in Shift Workers Improves Sleep and Reduces Circadian Disruption. Curr Biol.

[36] Bragard I, Coucke PA (2013). Impact of the use of Luminette® on well-being at work in a radiotherapy department. Cancer Radiother.

[37] Boivin DB, James FO (2005). Light treatment and circadian adaptation to shift work. Ind Health.

[38] Purnell MT, Feyer AM, Herbison GP (2002). The impact of a nap opportunity during the night shift on the performance and alertness of 12-h shift workers. J Sleep Res.

[39] de Assis MAA, Kupekb E, Nahasc MV (2003). Food intake and circadian rhythms in shift workers with a high workload. Appetite..

[40] Atkinson G, Fullick S, Grindey C, Maclaren D, Waterhouse J (2008). Exercise, energy balance and the shift worker. Sports Med.

[41] Patel S, Kon SSC, Nolan CM (2018). The Epworth Sleepiness Scale: Minimum Clinically Important Difference in Obstructive Sleep Apnea. Am J Respir Crit Care Med.

[42] Vercoulen JH, Swanink CM, Fennis JF, Galama JM, van der Meer JW, Bleijenberg G (1994). Dimensional assessment of chronic fatigue syndrome. J Psychosom Res.

[43] Vercoulen JH, Alberts M, Bleijenberg G (1999). De Checklist Individual Strength (CIS). Gedragstherapie..

[44] Worm-Smeitink M, Gielissen M, Bloot L (2017). The assessment of fatigue: Psychometric qualities and norms for the Checklist individual strength. J Psychosom Res.

[45] Beurskens AJ, Bültmann U, Kant I, Vercoulen JH, Bleijenberg G, Swaen G (2000). Fatigue among working people: validity of a questionnaire measure. Occup Environ Med.

[46] Chung F, Yegneswaran B, Liao P, Chung SA, Vairavanathan S, Islam S (2008). STOP questionnaire: a tool to screen patients for obstructive sleep apnea. Anesthesiology..

[47] Horne JA, Östberg O (1976). A self-assessment questionnaire to determine morningness-eveningness in human circadian rhythms. Int J Chronobiol.

[48] Bultmann U, de Vries M, Beurskens AJ, Bleijenberg G, Vercoulen JH, Kant I (2000). Measurement of prolonged fatigue in the working population: determination of a cutoff point for the checklist individual strength. J Occup Health Psychol.

[49] Johns MW (1991). A new method for measuring daytime sleepiness: the Epworth sleepiness scale. Sleep..

[50] Aaronson NK, Muller M, Cohen PD, Essink-Bot ML, Fekkes M, Sanderman R (1998). Translation, validation and norming of the Dutch language version of the SF-36 health survey in community and chronic disease populations. J Clin Epidemiol.

[51] Ware JE, Kosinski M, Gandek B (2005). SF-36 health survey manual and interpretation guide.

[52] Buysse DJ, Reynolds CF, Monk TH, Berman SR, Kupfer DJ (1989). The Pittsburgh Sleep Quality Index: a new instrument for psychiatric practice and research. Psychiatry Res.

[53] Parloff MB, Kelman HC, Frank JD (1954). Comfort, effectiveness, and self-awareness as criteria for improvement in psychotherapy. Am J Psychiatry.

[54] Kleijn WC, Hovens JE, Rodenburg JJ, Rijnders RJ (1998). Psychiatrische symptomen bij vluchtelingen aangemeld bij het psychiatrisch centrum De Vonk. Ned Tijdschr Geneeskd.

[55] Derogatis LR, Lipman RS, Rickels K, Uhlenhuth EH, Covi L (1974). The Hopkins Symptom Checklist (HSCL): a self-report symptom inventory. Behav Sci.

[56] Nabbe P, Le Reste JY, Guillou-Landreat M, Gatineau F, Le Floch B, Montier T (2019). The French version of the HSCL-25 has now been validated for use in primary care. PLoS One.

[57] Kleijn WC, Hovens JE, Rodenburg JJ. Posttraumatic stress symptoms in refugees: assessments with the Harvard Trauma Questionnaire and the Hopkins symptom Checklist-25 in different languages. Psychol Rep. 2001;88(2):527-32. 10.2466/pr0.2001.88.2.527 11351903

[58] Torquati L, Mielke GI, Brown WJ, Kolbe-Alexander T (2018). Shift work and the risk of cardiovascular disease. A systematic review and meta-analysis including dose-response relationship. Scand J Work Environ Health.

[59] Puttonen S, Härmä M, Hublin C (2010). Shift work and cardiovascular disease – pathways from circadian stress to morbidity. Scand J Work Environ Health.

[60] Puttonen S, Viitasalo K, Härmä M (2011). Effect of shiftwork on systemic markers of inflammation. Chronobiol Int.

[61] Faul F, Erdfelder E, Buchner A, Lang AG. Statistical power analyses using G*Power 3.1: tests for correlation and regression analyses. Behav Res Methods. 2009;41(4):1149–60. 10.3758/BRM.41.4.1149 19897823

