# Supplementary figures and images for: SHIFTPLAN: a randomized controlled trial investigating the effects of a multimodal shift-work intervention on drivers’ fatigue, sleep, health, and performance parameters
Source: Trials. 2022 Aug 17;23:662. doi: 10.1186/s13063-022-06573-6 (PMC9382013; doi:10.1186/s13063-022-06573-6)

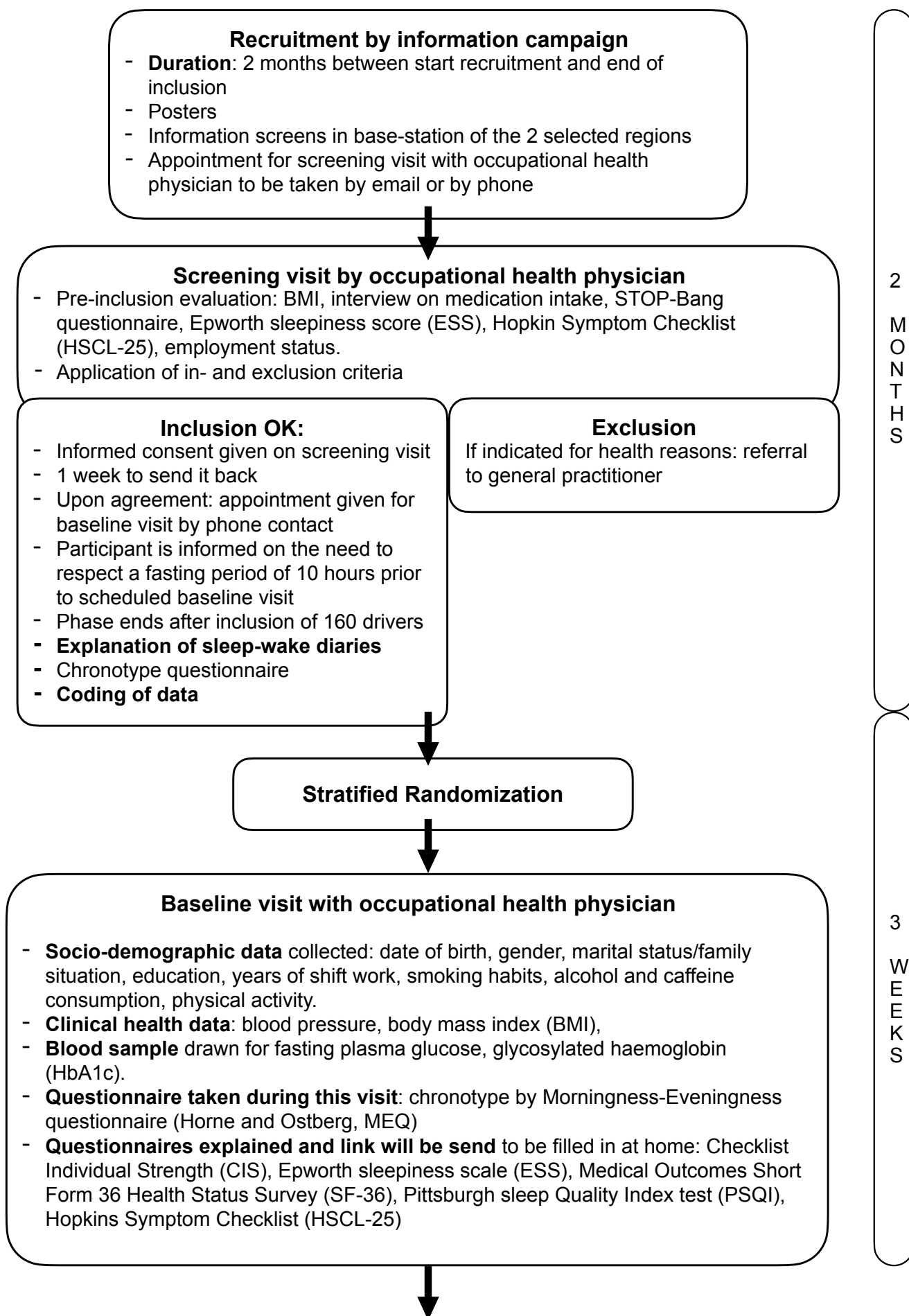

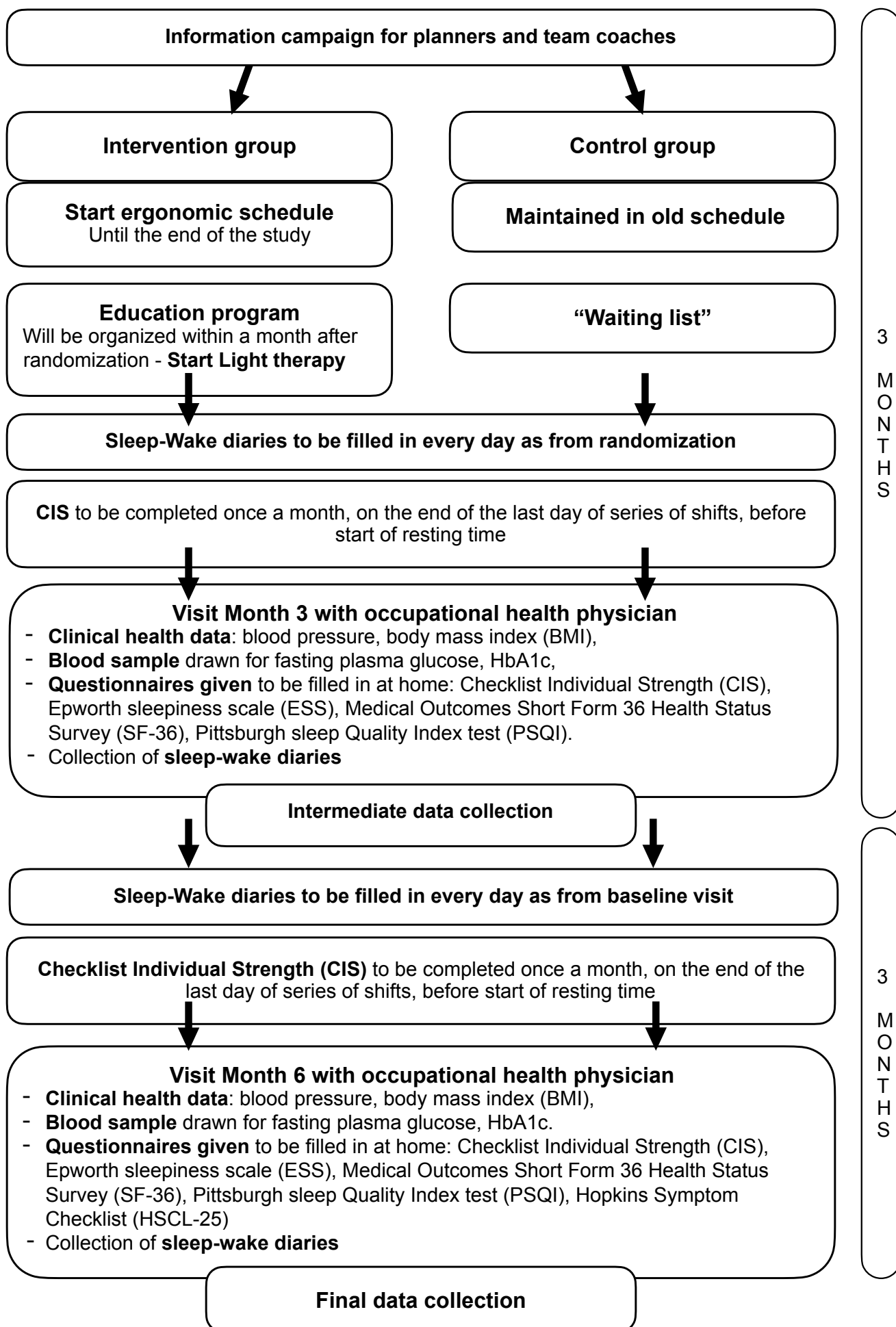

Supplement: Supplementary file 1 — Additional file 1. [file 13063_2022_6573_MOESM1_ESM.pdf]
