# Supplementary material for: SHIFTPLAN: a randomized controlled trial investigating the effects of a multimodal shift-work intervention on drivers’ fatigue, sleep, health, and performance parameters
Source: Trials. 2022 Aug 17;23:662. doi: 10.1186/s13063-022-06573-6 (PMC9382013; doi:10.1186/s13063-022-06573-6)
Supplement: Supplementary file 2 — Additional file 2. [file 13063_2022_6573_MOESM2_ESM.pdf]

STATISTICAL ANALYSIS PLAN

SHIFTPLAN

A randomized controlled trial investigating the effects of a multimodal shift-work intervention on drivers' fatigue, sleep, health and performance parameters.

Based on protocol version 14

Version: 3
Date: July 18th 2022

Statistician: Ella Roelant
Date:
Signature:

Principal Investigator: Inge Declercq
Date:
Signature:

Contents

List of Abbreviations 4
1. Introduction 5
2. Study objectives 5
2.1 Primary objective 5
2.2 Secondary objectives 5
2.3 Tertiary objective .... **Fout! Bladwijzer niet gedefinieerd.**
2.4 Exploratory objective 5
3. Study design 6
3.1 Overview 6
3.2 Study population 6
3.3 Sample size 6
3.4 Randomization 7
3.5 Study schedule 8
3.6 Study duration 11
4. Study endpoints 11
4.1 Primary endpoint 11
4.2 Secondary endpoint 11
4.3 Tertiary/safety endpoint 11
5. Sequence of planned analyses 11
5.1 Interim analyses 12
5.2 Final analyses and reporting 12
6. Statistical methods 12
6.1 Analysis principles 12
6.2 Incomplete follow-up, missing data and outliers 12
6.3 Data transformations 13
6.4 Multicenter study 13
6.5 Multiple comparisons and multiplicity 13
6.6 Data management and analysis software 13
7. Statistical analyses 13
7.1 Patient characteristics and baseline comparisons 13

7.2 Analysis of the primary endpoint 14
7.3 Analysis of secondary endpoint 14
7.4 Analysis of safety endpoints 15
7.5 Exploratory analyses 15
8. Reporting Conventions 15

List of Abbreviations

BANG: BMI, Age, Neck circumference, Gender
BMI: Body Mass Index
CIS: Checklist Individual Strength
ESS: Epworth Sleepiness Scale
HbA1c: glycosylated haemoglobin
HSCL-25: Hopkins Symptom Checklist
MEQ: Morningness-Eveningness Questionnaire
OSAS: Obstructive Sleep Apnea Syndrome
PI: Principal Investigator
PSQI: Pittsburgh Sleep Quality Index test
SD: standard deviation
SE: Sleep Efficiency
SF-36: Short Form 36 health status survey
STOP: Snoring, Tiredness, Observed apnea and high blood Pressure
TST: Total Sleep Time

1. Introduction

SHIFTPLAN is an open-label, multicenter, prospective, two arm 1:1 randomized comparative trial comparing an interventional arm where multiple shiftwork management measures are implemented namely

- healthy scheduling taking into account rotation, chronotype, resting time and napping;
- education program developed specifically for shift workers
- information campaign for the shiftwork planners

to a control arm who will continue to work in the old schedule and receive an information brochure. The study population are public transport bus drivers working as shift workers and employed by a public transport company.

For full references we refer to the protocol.

2. Study objectives

2.1 Primary objective

To evaluate whether a 6-month implementation of a multimodal shift-work management program will help ameliorate the symptomatic burden (i.e., improve sleepiness indices as measured with Epworth sleepiness scale (ESS)) in professional public transport drivers working shifts. The intervention will be considered successful if superiority is proven in this outcome.

2.2 Key secondary objective

To examine whether the intervention has an effect on fatigue indices (as measured by the Checklist Individual Strength, CIS) of the drivers.

2.3 Supplementary secondary objectives (tertiary objective)

To examine if the intervention has an effect on other parameters namely:

- Health-related quality of life
- Absenteeism
- Sleep outcomes
- Clinical health outcomes (bioclinicals)
- Mood and anxiety

2.4 Exploratory objective

The collected data will also be used to evaluate the outcomes in function of the chronotype of the participants.

3. Study design

3.1 Overview

A multicenter, two arm 1:1 randomized, open-label comparative trial evaluating an intervention with multiple shiftwork management measures versus control in professional drivers working as shift workers.

3.2 Study population

The study population are professional drivers working as shift workers and employed by a public transport company.

3.2.1 Inclusion criteria

- Public transport bus driver
- Working full time or 80% in the “old” (= habitual, backward rotating) schedule for at least 2 years

3.2.2 Exclusion criteria

- Regular medication for high blood pressure, diabetes, or for sleep disorder or medication for depression. Because secondary outcomes imply the evolution of blood pressure and blood sugar parameters, drivers with pre-existing comorbidity on these conditions, can not be found eligible for this study.
- High risk for moderate OSAS as evaluated by a STOP-Bang score of 6 or higher
- Drivers combining their employment as bus driver with another job
- Excessive daytime sleepiness: a high ESS above 12. Habitual cutoff is >9. Rationale for using a slightly higher cutoff is the fact that we will examine the effect of the study on daytime sleepiness. The applicants with an ESS >12 will be referred to a general practitioner for further evaluation.
- BMI higher than 35. The choice of this cut-off is determined according to existing data of the occupational health service of the company. In 2018, 42.6% of the drivers had a mean BMI of 25-30, and 27.5% had a BMI between 30-40.
- Presence of major depression as defined by a score above a threshold of 1.75 on the Hopkin Symptom Checklist (HSCL-25). This score was demonstrated to be highly suggestive of Major Depression, being defined as "a case requiring treatment”.

3.3 Sample size

For the primary and key secondary research question, we will be looking at two outcome measures, namely change in sleepiness (ESS) and in fatigue (CIS), both from baseline to the 6-month endpoint. We expect the outcomes for the intervention group to be superior to those obtained in the control group.

With regard to ESS, Patel et al. (2018) mentioned 2 or 3 units as being indicative of a clinical minimally important difference in obstructive sleep apnea. Viitasalo et al. (2008) found the largest SD for the ESS to be 5.3 units. Assuming a standard deviation of 5.3 and a significance

level of 0.05, an achieved sample size of 72 per group is required to detect an effect of 2.5 with 80% power using a two-sample t-test. Taking into account at least 20% drop-out, 88 drivers will be recruited into each group. Hence, a total of **176** drivers will be recruited for the study.

As to the CIS, we will consider a drop of 10 units to reflect a clinical minimally important difference (CMID). This number is in part based on Vercoulen et al. (1994) who considered scores of 27 or higher to indicate abnormal fatigue and scores ≥35 severe fatigue, where a drop of 8 or more units signifies a change in severity classification. Additionally, we refer to Worm-Smeitink et al. (2017) who, comparing CIS scores for breast cancer survivors and healthy controls in 2017, observed a 10-unit difference between the study population and the controls, with the former reporting higher levels of fatigue. We are thus confident that a difference of 10 units denotes a clinically relevant change.

For the standard deviation, we rely on Beurskens et al. (2000) who reported an SD of 18.9 units for a blue-collar population. Assuming a standard deviation of 19 and a significance level of 0.05 an achieved sample size of 58 per group would be required to detect an effect of 10 with 80% power using a two-sample t-test. Taking into account at least 20% drop-out, 70 drivers should be recruited into each group.

Based on these findings, we will adopt a minimum of 70 for each group in our study. Taking into account a drop-out of 20%, we will be recruiting 88 drivers per group (i.e., 176 drivers overall) to achieve this. Hence, with a total recruitment of 176 drivers, the study is sufficiently powered for both primary and key secondary outcomes. A power of 80% was a compromise as we needed the recruitment to be feasible and still be able to achieve sufficient power.

3.4 Randomization

To randomize the bus drivers 1:1 to control or intervention group, stratified randomization will be used. Stratification will be done according to region (2 regions), age (less than 40 years, 40 years or older), gender and BMI (BMI 25 and lower; BMI 26-30; BMI 31-35). The stratified randomization will be done by Qminim a web-based randomization system which uses minimization.

Minimization assures similar distribution of selected participant factors between study groups. The first participant is truly randomly allocated; for each subsequent participant the imbalance score is computed based on all previous allocations as well as the hypothetical allocation of the current patient to each treatment. The preferred treatment is then selected by choosing the treatment allocation associated with the smallest imbalance score.

The distance measure used to calculate the imbalance score is marginal balance. Marginal balance computes the cumulative difference between every possible pairs of level counts (i.e. the number of patients in that particular factor level).

The allocation of the new patient is then made at random with a heavy weighting in favour of the intervention that would minimize imbalance (a probability of 0.7 will be used).

3.5 Study schedule

	Screening (m-2,w-3,d-1)	Baseline (w-3)	d-1	0	1m	3m	6m
medication intake	x						
employment status	x						
history of diabetes	x						
history of depression	x						
Informed consent	x						
**Socio-demographic data**							
data of birth		x					
gender		x					
marital status/family		x					
education		x					
years of shift work		x					
smoking habits		x					
alcohol and caffeine consumption		x					
physical activity		x					
**Clinical health data**							
blood pressure		x				x	x
BMI	x	x				x	x
**Blood sample**							
fasting plasma glucose		x				x	x
HbA1c		x				x	x
Randomisation			x				
Start intervention				x			
**Questionnaires**							
MEQ for chronotype		x					
STOP-BANG	x						
CIS		x			x (monthly)	x	x
ESS	x	x				x	x
SF-36		x				x	x
PSQI		x				x	x
HSCL-25	x	x				x	x
Absenteeism							x (collection for whole period)
Qualitative assessment of implementation							x
**sleep wake diaries**		daily			daily	daily	daily
sleep-wake habits		daily			daily	daily	daily
sleep-wake schedules		daily			daily	daily	daily
mean total sleep time (TST)		daily			daily	daily	daily
sleep efficiency (SE)		daily			daily	daily	daily

All of the participants will be examined by an occupational health physician prior to the study (screening visit), at baseline and on 2 scheduled follow-up moments.

Evaluations:

1. Screening
2. Baseline
3. Outcomes 3 months after start of the program
4. Outcomes 6 months after start of the program

Screening

The applicants will be received for screening by the occupational health physician of their section.

Upon this screening visit, they will be informed again on all the aspects of the study, pre-inclusion evaluation will be done by the physician: BMI, interview on medication intake, STOP-Bang questionnaire, Epworth sleepiness score (ESS), Hopkin Symptom Checklist (HSCL-25), employment status. In- and exclusion criteria will be checked.

When inclusion is concluded possible and upon agreement of the driver, he/she will receive the informed consent and a have a week to sign it and send it back.

Baseline intake visit and randomization

The following socio-demographic data will be collected at baseline: date of birth, gender, marital status/family situation, education, years of shiftwork, smoking habits, alcohol and caffeine consumption, physical activity.

The following clinical health data will be collected by the external occupational health service: blood pressure, body mass index (BMI), fasting plasma glucose, glycosylated haemoglobin (HbA1c), high-sensitive C-reactive protein (hsCRP).

These data will be collected by an occupational health physician and trained nurse (to draw the blood samples). Fasting plasma glucose, glycosylated haemoglobin (HbA1c) will be taken after a fasting period of 10 hours. All of the clinical visits will be scheduled between 8 and 10 am on a day shift. If the participant has not respected the ten hour fasting period preceding this visit, another visit will be planned within a week with the trained nurse to draw the blood sample.

The following questionnaires will be administered for the collection of quantitative and qualitative data:

1. Checklist Individual Strength (CIS)
2. Epworth sleepiness scale (ESS)
3. Medical Outcomes Short Form 36 Health Status Survey (SF-36)
4. Pittsburgh sleep Quality Index test (PSQI)
5. Hopkins Symptom Checklist (HSCL-25)
6. Morningness-Eveningness questionnaire (Horne and Ostberg, MEQ)

All participants will receive these questionnaires at this intake visit and will be asked to return them within one week, except for the Morningness-Eveningness questionnaire to evaluate the chronotype (which the physician will have filled in during this visit).

At this visit, they will be thoroughly informed on how to fill in each questionnaire and on how to keep the sleep-wake schedules. The informed consent will include a paragraph to engage the participants to thoroughly follow all the directives (as keeping the sleep-wake diary on a daily basis) throughout the study.

Sleep-wake schedules will be used to collect qualitative data on sleep-wake habits and sleep-wake schedules, mean total sleep time (TST) and sleep efficiency (SE).

Randomization: After this baseline-intake visit, data of the drivers that have agreed to participate will be coded and these coded data will be sent to the principal investigator (PI).

Coding will be done by the occupational health physician and the codes will imply: region (2 regions), age (less than 40 years, 40 years or older), gender, BMI (BMI 25 and lower; BMI 26-30; BMI 31-35).

A code added for chronotype (divided into 3 groups) will be necessary since the participants randomized to the intervention group, will be assigned to a rotation schedule adapted to their chronotype.

The stratified randomization will then be done by Qminim a web-based randomization system. The participant will be randomized to intervention or to control group.

Measurements at 3 and 6 months follow-up

Following clinical data will be collected the external occupational health physician: blood pressure, BMI, blood sample for fasting plasma glucose, glycosylated haemoglobin (HbA1c), hsCRP.

Following questionnaires will be administered:

1. Checklist Individual Strength (CIS)
2. Epworth sleepiness scale (ESS)
3. Medical Outcomes Short Form 36 Health Status Survey (SF-36)
4. Pittsburgh sleep Quality Index test (PSQI)
5. Hopkins Symptom Checklist (HSCL-25)

The participants will receive the questionnaires at each follow-up and will be asked to return them within one week.

Furthermore, fatigue will be monitored on a more regular basis. Participants will be asked to fill in the fatigue scale (CIS)

- At baseline
- Once a month during the whole intervention, on the end of the last day of series of shifts, before start of resting time.

Sleep-wake schedules will be used to collect qualitative data on sleep-wake habits and sleep-wake schedules, mean sleep duration and sleep efficiency.

Measurement of absenteeism (data provided by the transport company): sick-leave data as expressed by number of sick-leave days will be available from official registrations.

Qualitative assessment of implementation and proximal effect of the intervention (change process) by short interview provided to both groups at the end of the study.

Questions:

1. To what extent are you satisfied with your well-being at the end of this study? (7-point Likert ranging from extremely satisfied to extremely in-satisfied)

2. If your experience was positive, why?

3. If your experience was negative, why?

4. If you were in the intervention group, would you like to continue working in this schedule? If you were not, would you like to change to the new schedule?

3.6 Study duration

Start recruitment is postponed due to COVID-19 pandemic and related restrictions. Inclusion runs for 2 months and follow-up is 6 months.

A participant is considered to have completed the study in case any of the following applies:

- Completion of planned follow-up period
- Lost to follow-up
- Refusal to be followed-up
- Withdrawal of informed consent

Data that has been collected until the time of withdrawal of consent will remain in the study data base unless the participant explicitly asks that all available data is removed.

4. Study endpoints

4.1 Primary endpoint

Symptomatic burden (sleepiness)

- Change in sleepiness from baseline to 6 months: daytime sleepiness as measured by Epworth Sleepiness Scale (ESS)

4.2 Key secondary endpoint

- Change in fatigue from baseline to 6 months as measured by CIS (Checklist Individual Strength)

4.3 Supplementary secondary endpoints

- General health-related quality of life (as measured by the SF-36)

- Absenteeism: sick-leave data as expressed by number of sick-leave days will be available from official registrations by transport company

- Sleep outcomes: Total sleep time (TST) and sleep efficiency (SE) as recorded by sleep-wake schedules and as measured by Pittsburgh sleep Quality Index test (PSQI)

- Clinical health outcomes: blood pressure, BMI, Fasting blood glucose, glycosylated haemoglobin (HbA1c), hsCRP

- Mood and anxiety, measured by the Hopkin Symptom Checklist (HSCL-25)

- Qualitative assessment of implementation

5. Sequence of planned analyses

5.1 Interim analyses

No interim efficacy analyses were planned.

5.2 Final analyses and reporting

All final planned analyses are conducted only after all patients have completed the 6 month follow-up.

Any extra post-hoc exploratory analyses performed to provide support for planned analyses, but not mentioned in the SAP will be documented and reported in appendices and clearly marked as unplanned analyses in any publication.

6. Statistical methods

6.1 Analysis principles

Primary analysis will be according to intention-to-treat principle, patients will be analyzed as in the randomized treatment group.

The per-protocol population is the population who followed the education program and worked for 6 months in the new system.

Two-sided 5% significance levels will be used to identify statistically significant results. All confidence intervals reported will be 95% confidence intervals.

6.2 Incomplete follow-up, missing data and outliers

6.2.1 Missing outcome data

Information that is unavailable due to withdrawal of consent, will not be considered ‘missing’, and will not be included in the calculation of the percentage of missing data described above.

The proposed linear mixed model allows that subjects have missing values at certain time points as the model uses all available data points per subject. The missing value assumption of this model is Missing At Random which means that missing values can only be dependent on the observed responses which seems a reasonable assumption in this case.

6.2.2 Missing baseline covariates

Baseline covariates are assumed not to be missing.

6.2.3 Outliers

Outliers will be identified by examination of residual plots. Cases that stand out are further examined for possible influence on the results by comparing analyses with and without these cases. When there are discrepancies between both analyses, this will be reported.

6.3 Data transformations

Data transformations using natural logarithm, inverse or square root can be considered when model assumptions of normality and constant variance of the residuals are not met.

6.4 Multicenter study

There are 2 distinct regions in the study. There will be explored if there are regional differences and if so a random intercept for region can be added to the linear mixed model to see if this has an impact on results.

6.5 Multiple comparisons and multiplicity

For the primary endpoint we are interested in detecting a significant effect sleepiness (ESS) of the intervention. To correct for the fact that we will need 2 tests to answer this question we have corrected the significance level with a factor 2 to keep the overall type I error at 5%.

In the analysis a less conservative multiple testing correction will be used namely Holm.

If in the mixed model time is significant as fixed effect, the different time points will be compared post-hoc to see where the differences lie. For this a multiple testing correction will be used e.g. Tukey or Holm.

6.6 Data management and analysis software

Data will be collected in REDCap.

All analyses will be performed in SAS version 9.4 or higher, or R version 3.5.2 or higher or SPSS version 21 or higher.

7. Statistical analyses

7.1 Patient characteristics and baseline comparisons

Demographic and other baseline characteristics will be summarized by group. For categorical variables, frequencies and percentages will be reported. Where values are missing, percentages will be calculated for the available cases, and the denominator will be mentioned. Continuous

variables will be summarized as mean with standard deviation, or median with interquartile range whatever is appropriate.

In case there are variables where clinical differences are observed between intervention and control group there will be corrected for in the regression models.

The following screening and baseline information will be collected:

- Age
- Gender
- Education
- Years of shift work
- BMI
- Active Smoking
- Alcohol consumption
- Cafeine consumption
- Marital and family situation
- Physical activity
- Chronotype
- History of Diabetes (on screening)
- History of Depression (on screening)

7.2 Analysis of the primary and key secondary endpoint

The primary endpoint and key secondary outcomes (changes in sleepiness and in fatigue) will be analyzed using an independent samples t-test in the intention-to-treat population comparing the intervention to the control group. Besides this, a linear regression model will be used to model the change in fatigue and the change in sleepiness from baseline to six months, with group as predictor and taking into account possible confounders gender, age, years of shift work, diabetes, BMI and smoking status.

7.3 Analysis of supplementary secondary endpoints

- To evaluate the sensitivity of the results of the primary outcome analysis, linear regression will be used to model the change in fatigue (CIS) from baseline to 6 months, and the change in sleepiness (ESS) from baseline to 6 months, with group as predictor and taking into account possible confounders like gender, age, years of shift work, diabetes, BMI and smoking.
- We will consider also the per-protocol analysis where only drivers who follow the protocol will be used.
- As the fatigue and sleepiness are measured several times (CIS, monthly and ESS 3-monthly) we will also consider a linear mixed model with subject as a random effect to model their evolution over time. This model allows the correction of confounders and the difference between the groups can be estimated at the different time points.
- To compare the continuous outcomes (SF-36, PSQI, HSCL-25, absenteeism, clinical health outcomes) at 6 months between the two groups we will use an independent samples t-test or Mann Whitney U test (whatever is appropriate). We can also fit a linear regression model for these outcomes which makes it possible to correct for confounders.

- A linear mixed model will be studied for the continuous outcomes (sleep times, sleep efficiency, SF-36, PSQI, HSCL-25, clinical health outcomes) measured over time.
- For the CIS and SF-36 besides the total score we will also consider the subscales to see on which domain the intervention has the largest impact.
- To handle missing data, we will use a mixed model with multiple imputation by chained equations. The imputation procedure will include intervention, gender, age, years of shift work, diabetes, BMI and smoking status and the available CIS and ESS measurements, respectively. This will generate 20 completed datasets that will be analysed separately. The results will be pooled.
- Description of the qualitative data obtained by the sleep-wake schedules
- Description of the qualitative assessment of the implementation and proximal effect of intervention.

7.4 Analysis of safety endpoints

Not applicable

7.5 Exploratory analyses

- We are interested to see if the intervention effect is different depending on the chronotype hence an interaction between intervention and chronotype will be considered.
- We are interested in association between chronotype and other variables like BMI, mental health (as tested by the HSCL-25), fatigue scores (CIS), sleepiness scores (ESS), hence this will also be explored.

8. Reporting Conventions

For linear regression models and linear mixed effects models coefficients, standard errors and p-values will be reported. For logistic and Cox regression models respectively odds ratios and hazard ratios, together with 95% confidence intervals and p-values will be given.
