# Supplementary material for: SHIFTPLAN: a randomized controlled trial investigating the effects of a multimodal shift-work intervention on drivers’ fatigue, sleep, health, and performance parameters
Source: Trials. 2022 Aug 17;23:662. doi: 10.1186/s13063-022-06573-6 (PMC9382013; doi:10.1186/s13063-022-06573-6)
Supplement: Supplementary file 3 — Additional file 3. [file 13063_2022_6573_MOESM3_ESM.pdf]

UZA / Wilrijkstraat 10 / 2650 Edegem
Parking via Drie Eikenstraat 655
Tel +32 3 821 30 00
www.uza.be / BE0874.619.603

Universiteit Antwerpen

COMITE VOOR MEDISCHE ETHIEK

Dr. I. Declercq
Slaapcentrum

VOORZITTER
Prof. dr. Patrick Cras

SECRETARIAAT
tel: 03 821 38 97

Shift werk, slaap en gezondheid: effect van de implementatie van een veelzijdig shiftwerk interventie-programma op slaap-en gezondheidsparameters en absenteïsme van professionele buschauffeurs werkzaam in de publieke sector. (EDGE 000339)

Belgisch Registratienummer: B300201941122

datum	ons kenmerk	contact
19/08/2019	19/27/328	Secretariaat Ethisch Comité<br>ethisch.comite@uza.be

DEFINITIEF GUNSTIG ADVIES

Geachte Collega,

Het Ethisch Comité van het Universitair Ziekenhuis Antwerpen en de Universiteit Antwerpen bevestigt dat bovenvermelde studie voldoet aan de criteria gesteld in de wet van 7 mei 2004 en geeft een gunstig advies dd. 19/08/2019 .

De volgende bijlagen werden volgens de ICH-GCP richtlijnen door het Ethisch Comité goedgekeurd:

- CV Onderzoeker UZA
  Dr. I. Declercq, Prof. Dr. F. Van Den Eede
- Informatie- en toestemmingsformulier NL aangepaste versie dd. 22/07/2019
  versie 3 (Clean & Track Changes)
- Protocol aangepaste versie dd. 22/07/2019
  Version 8 (Clean & Track Changes)
- Protocol - Samenvatting aangepaste versie dd. 22/07/2019
  Version 8 (Clean & Track Changes)
- Vragenlijst(en) aangepaste versie dd. 22/07/2019
  1) CIS-20: Checklijst Individuele Spankracht, versie 2
  2) ESS: Epworth Sleepiness Scale, versie 2
  3) SF-36: IQOLA SF-36 Dutch (BE) Gezondheidstoestand vragenlijst, versie 2
  4) PSQI: Pittsburgh Slaap Kwaliteit Index, versie 2
  5) HSCL-25: Hopkin Symptom Checklist 25, versie 2
  6) MEQ: Ochtendtype-Avondtype vragenlijst van Horne en Ostberg, versie 2
  7) STOP-Bang vragenlijst, versie 2
  8) Voorbeeld slaap-waak dagboek / Invullen, versie 2
- Diverse
  SHIFTPLAN Contract De Lijn-UZA-IDEWE draft2

De volgende opmerkingen werden nog gemaakt:

1) Gelieve te vermelden dat het corrigeren van persoonsgegevens voor de deelnemers mogelijk is.
2) De studie kan pas opgestart worden als het contract definitief goedgekeurd is.

UZA / Wilrijkstraat 10 / 2650 Edegem
Parking via Drie Eikenstraat 655
Tel +32 3 821 30 00
www.uza.be / BE0874.619.603

Universiteit Antwerpen

datum	ons kenmerk	contact
19/08/2019	19/27/328	Secretariaat Ethisch Comité<br>ethisch.comite@uza.be

Deze goedkeuring is geldig tot een jaar na bovenvermelde datum. Wij verzoeken u ons te melden wanneer de eerste deelnemer werd geïncludeerd, wanneer en waarom de studie (vroegtijdig) werd stopgezet of nooit werd opgestart.
Indien de studie nog loopt na een jaar verwachten we een follow-up rapport waarin eventuele voorvallen worden gemeld.
Tot slot wijzen we er op dat, voor in het UZA lopende studies, de ernstige ongewenste voorvallen dienen gerapporteerd via het incidentenmeldingssysteem

Met vriendelijke groeten,

Prof. dr. P. Cras
Voorzitter Ethisch Comité

Cc: FAGG - Research & Development Department, Victor Hortaplein 40, bus 40 - 1060 Brussel
Prof. dr. F. Van Den Eede, UZA - Psychiatrie - 2650 Edegem
Prof. dr. J. Verbraecken, UZA - Slaapcentrum - 2650 Edegem

UZA / Wilrijkstraat 10 / 2650 Edegem
Parking via Drie Eikenstraat 655
Tel +32 3 821 30 00
www.uza.be / BE0874.619.603

Universiteit Antwerpen

datum	ons kenmerk	contact
19/08/2019	19/27/328	Secretariaat Ethisch Comité<br>ethisch.comite@uza.be

Samenstelling Ethisch Comité sinds 7/01/2019.
Deze studie werd besproken op vergadering van 19/08/2019.

	Functie	M/V	Aanwezig
Voorzitter			
CRAS Patrick	Voorzitter/Neuroloog	M	+
Ondervoorzitter			
IEVEN Greet	Ondervoorzitter/Klinisch Bioloog	V	+
Leden EC UZA			
BASTIAENS Valerie	Apotheker	V	-
BLAUMEISER Bettina	Medisch geneticus	V	+
DE BAETSELIER Elyne	Verpleegkundige	V	+
HENS Kristien	Ethicus	V	-
MICHIELS Barbara	Huisarts	V	-
MICHIELSEN Peter	Gastro-enteroloog	M	+
PAELINCK Bernard	Cardiochirurg	M	-
SPECENIER Pol	Oncoloog	M	+
VAN DE WIELE Miranda	Patiëntenbegeleiding	V	-
VAN DEN EEDE Filip	Psychiater	M	+
VAN DYCK Pieter	Radioloog	M	-
VANSWEEVELT Thierry	Jurist	M	-
VERLOOY Joris	Pediater	M	-
Toegevoegde leden UA			
BORTIER Hilde	Arts, emerita hoogleraar UA	V	+
DE MEESTER Ingrid	Farmacoloog / Onderzoeker UA	V	-
GRANAAS Kristina	Medewerkster Dep. Onderzoek	V	+
Uitgenodigde Experten			
AERTSEN Veerle	Patiëntenvertegenwoordiger	V	-
DOCKX Celine	Jurist	V	-
IDES Kris	Kinesitherapeut	M	+
LUYTEN Leon	Arts in het beheer van gezondheidsgegevens	M	+
MICHIELSENS Inge	Jurist	V	-
MOONS Pieter	Coördinator bio- en weefselbank	M	-
VAN BORTEL Paulus	Filosoof	M	-
VANDEPERRE Peggy	Vertegenwoordigster gezonde vrijwilligers	V	-

The Ethics Committee states that no individual member of the Ethics Committee who may have an affiliation with the study or sponsor, has voted in the deliberations for this trial.

The Ethics Committee states that it is organised and operates according to the ICH/GCP guidelines, the applicable laws and regulations, and their own written operating procedures.
